# Autologous Tissue Grafts for Chin Augmentation With or Without Genioplasty: A Systematic Review

**DOI:** 10.3390/jcm15187025

**Published:** 2026-09-10

**Authors:** Kamil Nelke, Agnieszka Kotela, Zuzanna Majchrzak, Marzena Laszczyńska, Tomasz Horodniczy, Kamil Wesołek, Agata Małyszek, Jacek Matys, Maciej Dobrzyński

**Affiliations:** 1 Maxillo-Facial Surgery Ward, EMC Hospital, Pilczycka 144, 54-144 Wroclaw, Poland; kamil.nelke@gmail.com; 2 Academy of Applied Sciences, Health Department, Academy of Silesius in Wałbrzych, Zamkowa 4, 58-300 Wałbrzych, Poland; 3 Medical Center of Innovation, Wroclaw Medical University, Krakowska 26, 50-425 Wroclaw, Poland; kotela.agnieszka@gmail.com (A.K.); zuzanna.h.nawrocka@gmail.com (Z.M.); tomasz.horodniczy2@gmail.com (T.H.); 4 Józef Struś Multi-Specialty Municipal Hospital with a Care and Treatment Facility, Independent Public Health Care Facility, Szwajcarska 3, 61-285 Poznan, Poland; wesolekwesolek@o2.pl; 5 Department of Biostructure and Animal Physiology, Wrocław University of Environmental and Life Sciences, Kożuchowska 1, 51-631 Wroclaw, Poland; 6 Dental Surgery Department, Wroclaw Medical University, Krakowska 26, 50-425 Wroclaw, Poland; 7 Department of Pediatric Dentistry and Preclinical Dentistry, Wroclaw Medical University, Krakowska 26, 50-425 Wroclaw, Poland; maciej.dobrzynski@umw.edu.pl

**Keywords:** autologous graft, genioplasty, chin augmentation, chin advancement, bone graft, adipose tissue, cartilage, dermal tissue, systematic review

## Abstract

**Objective:** This systematic review evaluated the clinical application of autologous tissue grafts for chin augmentation performed with or without genioplasty. The primary outcomes included clinical and aesthetic improvement, graft stability and integration, resorption, complications, patient satisfaction, and the need for secondary procedures. **Methods:** The review was prospectively registered in OSF and conducted in accordance with the PRISMA 2020 statement. PubMed, Scopus, Embase, Web of Science, and WorldCat were searched using terms related to genioplasty, chin advancement, and autologous grafting materials, including bone, adipose tissue, cartilage, dermal tissue, and tooth-derived grafts. Eligible studies included original clinical publications involving human patients and reporting outcomes following chin augmentation with an autologous tissue graft, with or without genioplasty. Study selection and data extraction were conducted independently according to predefined eligibility criteria. Methodological quality was assessed using the appropriate Joanna Briggs Institute critical appraisal tools. Because of substantial clinical and methodological heterogeneity across the included studies, no meta-analysis was performed, and the findings were instead synthesized qualitatively. **Results:** Seventeen publications were included, comprising predominantly retrospective studies, case series, case reports, and technique-oriented clinical reports, with only one prospective randomized comparative trial; the overall level of evidence was therefore low, and comparative data across graft types remained limited. The evaluated materials comprised autologous adipose tissue, dermal grafts, iliac crest bone, costal cartilage and costochondral grafts, coronoid process bone, external oblique line corticocancellous bone, mandibular bone harvested during orthognathic surgery, a third-molar tooth graft, and an osteocartilaginous nasal hump graft. Most studies reported improvements in chin projection, facial profile, symmetry, or lower facial proportions. The available evidence suggests that autologous bone and cartilage grafts may provide integration and structural support, with limited clinically evident resorption reported; however, these observations derive from limited and heterogeneous evidence. Soft-tissue grafts improved chin contour but showed less predictable volume maintenance. Dermal graft resorption reached approximately 35% after 12 months, while fat grafting was associated with soft-tissue relapse and occasional secondary lipofilling. Serious graft-related complications were not frequently reported; however, adverse-event reporting was inconsistent, preventing reliable estimation of their incidence. Reported events included infections, temporary sensory disturbances, contour irregularities, and isolated graft removals. The certainty of the findings was limited by heterogeneous study designs, predominantly small or uncontrolled samples, variable outcome measures, and inconsistent follow-up. **Conclusions:** Autologous tissue grafts represent potentially effective options for chin augmentation when the graft source and surgical technique are selected according to the type and extent of the deformity. The available evidence suggests that bone and cartilage grafts may provide structural support, whereas adipose and dermal tissues may be considered for moderate soft-tissue augmentation; however, these conclusions are based on limited and heterogeneous evidence. However, the available evidence does not establish the superiority of autologous grafts over sliding genioplasty or alloplastic implants. Registration: Open Science Framework.

## 1. Introduction

**Figure 1:**
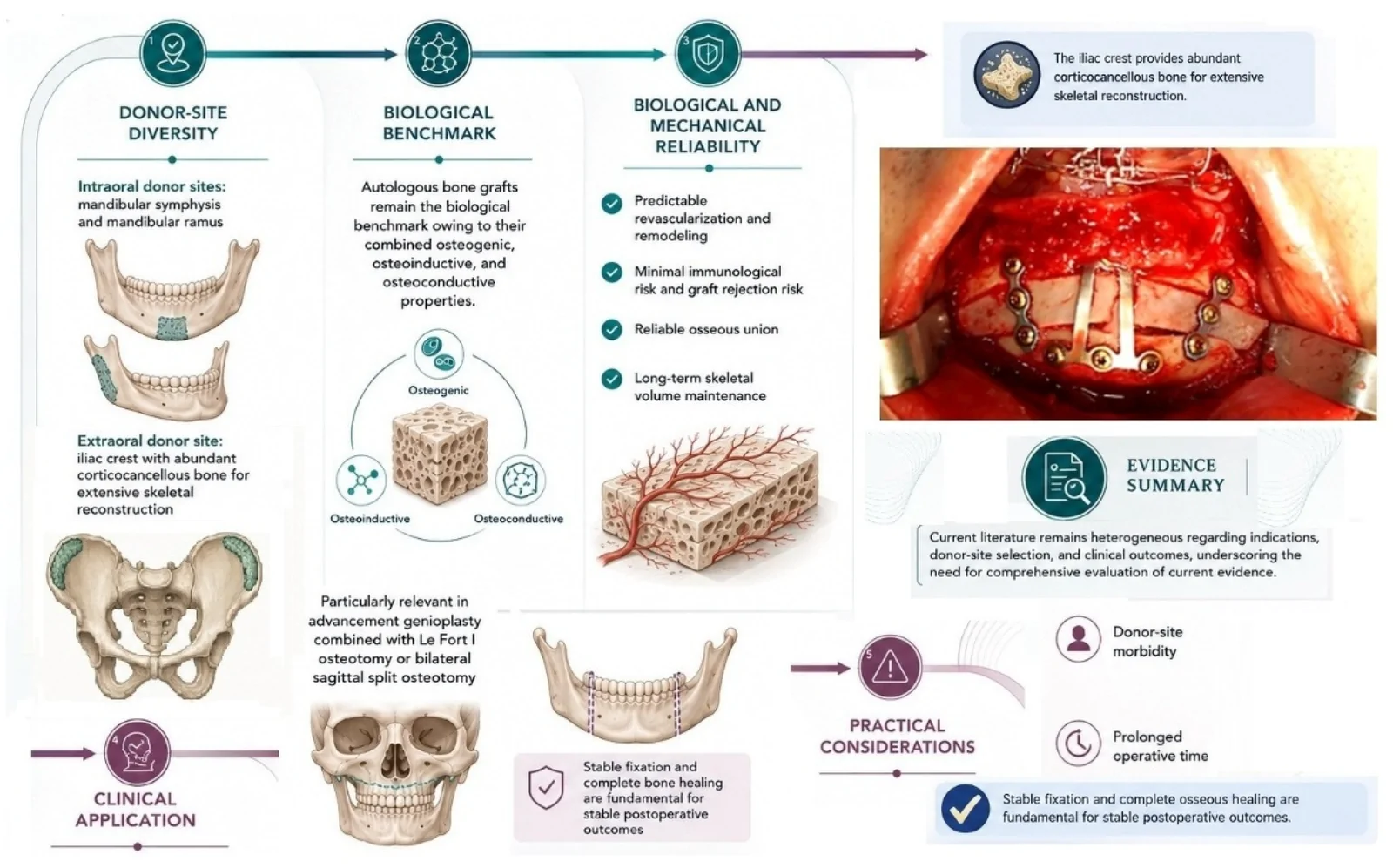
Clinical rationale for autologous bone grafting in advancement genioplasty: donor-site options, biological properties, mechanical reliability, and practical considerations.

Chin projection plays a key role in the aesthetics and function of the lower face by influencing the facial profile, vertical and horizontal facial proportions, and the perception of overall facial harmony [1,2]. Insufficient or excessive chin projection may create the appearance of a weak or, conversely, overly prominent lower facial contour, disrupting the relationship between the nose, lips, and chin and potentially causing considerable psychosocial discomfort [3]. From a clinical perspective, chin position and morphology are also closely related to lower-lip position, the depth of the labiomental fold, mentalis muscle tone, and the contour of the cervicomental angle, with potential implications for lip function, swallowing, and upper airway patency [4]. Several treatment modalities have been described, including sliding genioplasty, alloplastic chin implants, and different forms of autologous tissue grafting. Each approach has specific indications, advantages, and limitations, emphasizing the importance of selecting the most appropriate treatment according to the individual anatomical and clinical characteristics of the patient [1,5,6,7,8,9,10]. Genioplasty is a versatile and predictable procedure that enables simultaneous correction of chin projection in the sagittal and vertical planes, as well as modification of chin width and symmetry through repositioning of the bony segment, with or without additional augmentation using autologous grafts [11,12]. Depending on the type and extent of the deformity, different modifications may be applied, including conventional sliding genioplasty, T-genioplasty, single-piece segment lateralization, and chin-wing osteotomy [13,14]. In orthognathic treatment, genioplasty may complement maxillomandibular correction by enabling precise adjustment of the pogonion position following Le Fort I and bilateral sagittal split osteotomies and by improving the harmony of the lower facial third even after satisfactory dental and skeletal relationships have been achieved [15,16,17,18,19,20,21,22,23]. When carefully planned on the basis of cephalometric analysis, facial proportions, skeletal stability, and patient expectations, genioplasty can improve the mandibular contour, cervicomental profile, and overall facial balance [6,24] (see Figure 1).

Sliding advancement genioplasty is a well-established procedure for correcting chin retrusion, improving lower facial projection, and optimizing facial harmony, both as an isolated aesthetic procedure and as an adjunct to comprehensive orthognathic treatment. However, substantial advancement of the chin segment may create intersegmental osseous gaps and reduce the cortical bone contact area, potentially compromising mechanical stability and increasing the risk of relapse, delayed bone healing, or contour irregularities [24,25]. Although repositioning of the native chin segment is sufficient in many patients, additional augmentation may be required in cases involving extensive skeletal deficiency, marked asymmetry, vertical or transverse deficiency, or complex dentofacial deformity. Osseous gaps or residual contour deficiencies may be managed using autologous, allogeneic, or xenogeneic grafts, depending on the size and location of the defect and the requirements of the planned surgical procedure [26,27,28,29,30,31,32,33,34,35]. Alternative approaches include alloplastic implants and individually designed patient-specific devices. Despite their availability and the absence of donor-site morbidity, non-autologous materials may be associated with biological and mechanical complications, including infection, soft-tissue dehiscence, foreign-body reaction, implant displacement, bone resorption, or inadequate osseointegration [36]. Furthermore, graft substitutes may not always provide the structural support and biological environment required for predictable healing following extensive chin advancement [37]. Consequently, reconstruction of osseous deficiencies during genioplasty remains an important clinical challenge, particularly in patients requiring advancement exceeding 10 mm or genioplasty combined with other orthognathic procedures, in whom long-term skeletal stability is essential for maintaining both functional and aesthetic outcomes [24,26].

Among the available reconstructive options, autologous bone remains an important reference material because it combines osteogenic, osteoinductive, and osteoconductive properties. Depending on the volume and geometry of the defect, grafts may be harvested from intraoral donor sites, such as the mandibular symphysis or ramus [29], or from extraoral sites, most commonly the iliac crest, which provides a relatively large volume of corticocancellous bone for extensive skeletal reconstruction [38]. Autologous bone can undergo revascularization, incorporation, and subsequent remodeling within the recipient site while avoiding the risk of immunological incompatibility associated with non-autologous materials. These properties may be particularly relevant when advancement genioplasty is combined with Le Fort I osteotomy or bilateral sagittal split osteotomy, where stable fixation, restoration of osseous continuity, and predictable bone healing are essential for maintaining postoperative skeletal stability [16,17,18,24]. An iliac crest corticocancellous graft may be particularly useful when a substantial volume of bone is required, including extensive chin advancement or combined maxillary and mandibular repositioning [38,39,40,41,42]. Nevertheless, autologous bone harvesting may increase operative time and may be associated with donor-site pain, morbidity, scarring, or other site-specific complications. The decision to use an autologous graft should therefore consider the extent and location of the defect, the required graft volume, the availability of an appropriate donor site, patient-related factors, and the expected balance between biological benefits and donor-site morbidity.

Despite the wide range of available surgical techniques and augmentation materials, the clinical evidence concerning autologous grafting in the chin region remains fragmented. Most available studies evaluate individual techniques, include small and heterogeneous patient groups, or focus on specific clinical indications, thereby limiting direct comparisons between treatment modalities and complicating the formulation of evidence-based recommendations [3,5,38]. The diversity of surgical procedures, grafting materials, and patient-specific factors further complicates the selection of an optimal treatment strategy [14,26,27,28,29]. Autologous tissue grafts used for chin augmentation include bone, adipose tissue, cartilage, dermal tissue, and other patient-derived materials. These grafts may be applied independently or in conjunction with sliding genioplasty, depending on the primary treatment objective. Structural grafts, particularly bone and cartilage, may provide structural augmentation, with bone grafts also used to restore osseous continuity when required. In contrast, adipose and dermal grafts primarily modify soft-tissue volume and contour and do not themselves provide skeletal advancement. However, no contemporary systematic synthesis has comprehensively evaluated these different autologous grafts in terms of long-term stability, graft integration and resorption, aesthetic and functional outcomes, complications, patient-reported outcomes, and the need for secondary procedures [1,6,38]. A structured synthesis of these clinically distinct autologous approaches is therefore clinically relevant, as it may improve patient selection, facilitate individualized surgical planning, and support graft selection according to the underlying anatomical deficiency, reconstructive objective, and surgical context [6,15,24,26].

Therefore, the aim of the present systematic review was to evaluate the available clinical evidence concerning the use of autologous tissue grafts for chin augmentation performed with or without genioplasty. Particular emphasis was placed on the types and donor sites of autologous grafts, their clinical indications, surgical applications, long-term stability, integration and resorption, associated complications, patient-reported outcomes, and the need for secondary procedures. To the best of our knowledge, no contemporary systematic review has focused specifically on the full spectrum of autologous tissue grafts used for chin augmentation. Existing broader reviews of genioplasty, chin augmentation, and bone grafting in orthognathic surgery are not specifically focused on this clinical question and may not reflect recent developments in contemporary autologous grafting techniques [26].

## 2. Materials and Methods

### 2.1. Focused Question

The focused question was formulated according to the PICO framework [43]:

- Population (P): Patients undergoing chin augmentation, performed either as an isolated procedure or in combination with genioplasty.
- Intervention (I): Chin augmentation using autologous tissue grafts, including bone, adipose tissue, cartilage, dermal tissue, tooth-derived grafts, or composite autologous materials.
- Comparison (C): A comparator was not required for inclusion because many of the included studies were non-comparative (case series, case reports, and single-arm technique reports). Where a comparator was available, it consisted of chin augmentation performed without an autologous graft or using alternative techniques or non-autologous materials (e.g., sliding genioplasty alone or alloplastic implants), and such comparative data were considered during synthesis. However, the review was not designed to establish the superiority of autologous grafting over these alternative procedures; rather, its aim was to characterize the outcomes reported for autologous grafts across the available literature.
- Outcomes (O): Clinical and aesthetic outcomes, graft integration, long-term stability, graft resorption, functional outcomes, complications, patient satisfaction, and the need for secondary procedures.

Based on this framework, the following research question was formulated: In patients undergoing chin augmentation with or without genioplasty (P), what clinical outcomes have been reported following the use of autologous tissue grafts (I)—and, where available, in comparison with alternative techniques or non-autologous materials (C)—including graft stability, resorption, complications, patient satisfaction, and need for secondary procedures (O)? The review was intended to describe and synthesize these reported outcomes rather than to determine the comparative superiority of any single approach.

### 2.2. Protocol

**Figure 2:**
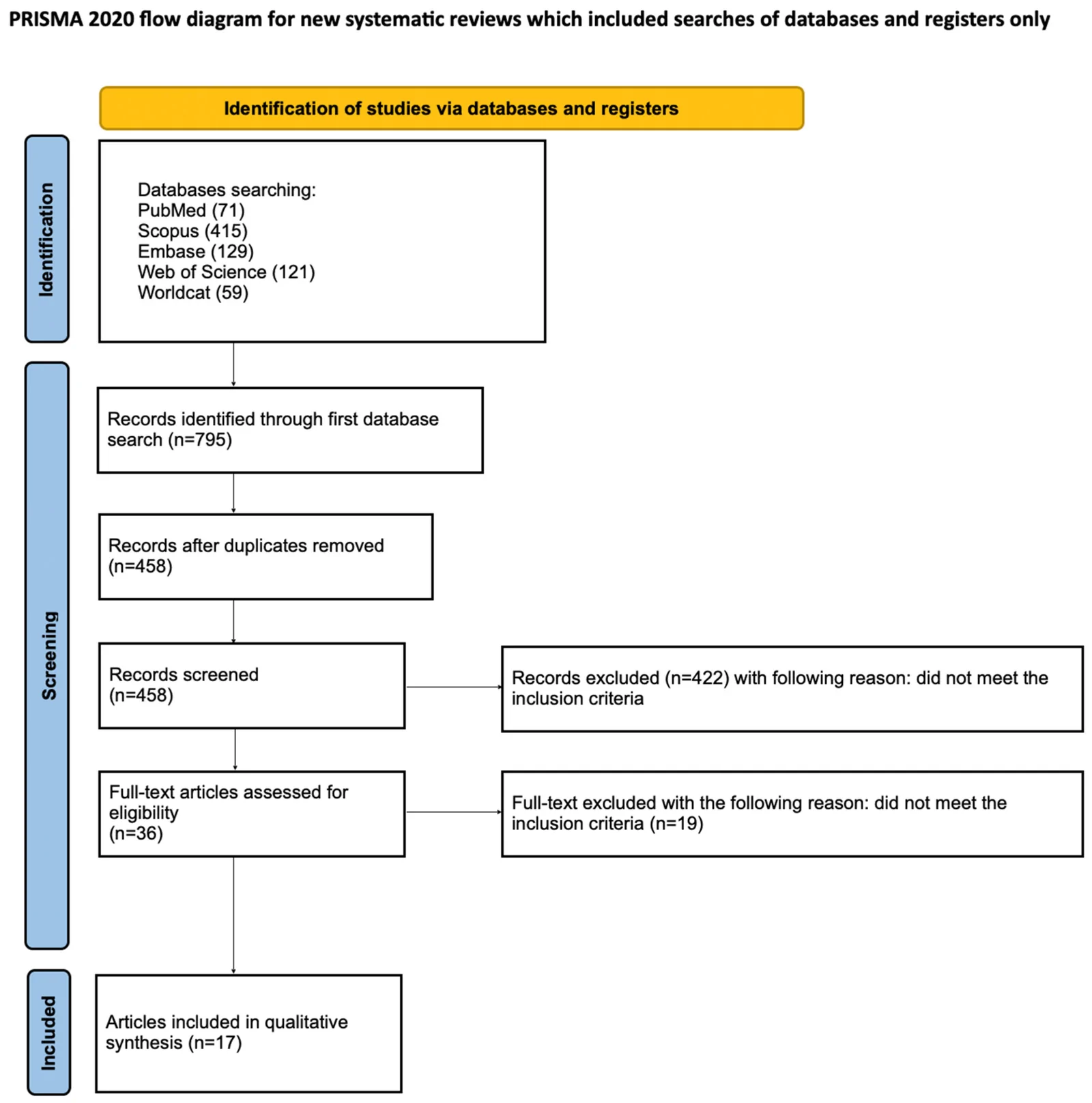
The PRISMA 2020 flow diagram.

This systematic review was conducted and reported in accordance with the Preferred Reporting Items for Systematic Reviews and Meta-Analyses 2020 statement (PRISMA 2020; Supplementary Table S1). The review protocol was prospectively registered in the Open Science Framework (OSF) before the formal study-selection process commenced. The complete protocol is available at: https://osf.io/8fgc9 (accessed on 23 July 2026). The consecutive stages of study identification, duplicate removal, title and abstract screening, full-text eligibility assessment, and final study inclusion are presented in the PRISMA 2020 flow diagram in Figure 2 [44].

### 2.3. Eligibility Criteria

Studies were considered eligible when they met all of the following inclusion criteria [45,46,47,48,49,50,51,52,53,54,55,56]:

- original clinical research, including randomized clinical trials, prospective or retrospective observational studies, comparative studies, case series, and case reports;
- studies involving human participants with chin deficiency, microgenia, insufficient chin projection, vertical chin deficiency, mandibular hypoplasia, retrognathia, post-traumatic deformity, or another condition requiring chin augmentation;
- application of an autologous tissue graft, including bone, adipose tissue, cartilage, dermal tissue, tooth-derived grafts, or composite autologous materials, for chin augmentation performed with or without genioplasty;
- reporting of original clinical, radiographic, aesthetic, functional, patient-reported, stability, resorption, or complication-related outcomes;
- availability of the complete article in English.

The presence of a control or comparison group was not required, because non-comparative studies, case series, and case reports were eligible for inclusion. Consistent with the PICO framework above, comparator data were extracted and considered whenever they were reported, but their absence did not preclude inclusion, and the review was not structured as a comparative-effectiveness analysis.

Studies were excluded for any of the following reasons: [S1-S19] (Supplementary Table S2).

- non-human or preclinical research;
- review articles, systematic reviews, meta-analyses, editorials, expert opinions, commentaries, letters without original patient data, or other non-original publications;
- conference abstracts, meeting proceedings, or poster presentations without an accompanying full-text article;
- publications not available in English;
- inability to obtain the complete article;
- absence of an autologous tissue graft used for chin augmentation;
- absence of original clinical outcomes relevant to the review;
- purely technical descriptions without patient-level outcome data;
- duplicate records or multiple publications reporting the same clinical population without additional relevant outcomes.

No restrictions were imposed on the year of publication.

### 2.4. Information Sources, Search Strategy, and Study Selection

A systematic literature search was conducted in five electronic databases: PubMed, Scopus, Embase, Web of Science Core Collection, and WorldCat. The search strategy combined terms related to genioplasty and chin advancement with terms describing autologous tissue grafting. Database-specific field tags, indexing terms, and controlled vocabulary were applied where available. No restrictions concerning the year of publication were used. The search was updated in July 2026, and no additional eligible studies were identified. The core search structure was identical across all databases and combined two concept blocks with the Boolean operator AND: a chin-procedure block (genioplasty, chin advancement, sliding genioplasty, chin osteotomy) and an autologous-graft block. The syntax of each strategy was then adapted to the indexing system, field tags, and controlled vocabulary of the individual database (e.g., [Title/Abstract] in PubMed, TITLE-ABS-KEY in Scopus, TS = in Web of Science, and/exp explosion of Emtree terms in Embase), which accounts for the differences in appearance among the queries. These differences reflect database-specific implementation of the same underlying concepts rather than differences in search scope, with one intentional exception: WorldCat, which was used only as a supplementary discovery source for grey literature and non-indexed records, employed a deliberately shorter query built on the core concepts, as its limited field-searching capability does not reliably support long tissue-specific term strings.

The search syntax was adapted to the indexing system and available search fields of each database. The following strategies were applied:
PubMed(“genioplasty”[Title/Abstract] OR “chin advancement”[Title/Abstract] OR “sliding genioplasty”[Title/Abstract] OR “chin osteotomy”[Title/Abstract]) AND (“iliac crest”[Title/Abstract] OR autogenous[Title/Abstract] OR autograft*[Title/Abstract] OR corticocancellous[Title/Abstract] OR “adipose tissue”[Title/Abstract] OR cartilage[Title/Abstract] OR “dermal tissue”[Title/Abstract] OR tooth[Title/Abstract])Scopus(TITLE-ABS-KEY(genioplasty) OR TITLE-ABS-KEY(“chin advancement”) OR TITLE-ABS-KEY(“sliding genioplasty”) OR TITLE-ABS-KEY(“chin osteotomy”)) AND (TITLE-ABS-KEY(“iliac crest”) OR TITLE-ABS-KEY(autogenous) OR TITLE-ABS-KEY(autograft*) OR TITLE-ABS-KEY(corticocancellous) OR TITLE-ABS-KEY(“adipose tissue”) OR TITLE-ABS-KEY(cartilage) OR TITLE-ABS-KEY(“dermal tissue”) OR TITLE-ABS-KEY(tooth))Web of ScienceTS = (genioplasty OR “chin advancement” OR “sliding genioplasty” OR “chin osteotomy”) AND TS = (“iliac crest” OR autogenous OR autograft* OR corticocancellous OR “adipose tissue” OR cartilage OR “dermal tissue” OR tooth)Embase(‘genioplasty’/exp OR genioplasty:ti,ab OR ‘chin advancement’:ti,ab OR ‘sliding genioplasty’:ti,ab OR ‘chin osteotomy’:ti,ab) AND (‘autologous bone graft’/exp OR ‘iliac crest’/exp OR ‘iliac crest’:ti,ab OR autogenous:ti,ab OR autograft*:ti,ab OR corticocancellous:ti,ab OR ‘adipose tissue’:ti,ab OR cartilage:ti,ab OR ‘dermal tissue’:ti,ab OR tooth:ti,ab)WorldCat(genioplasty OR “chin advancement” OR “sliding genioplasty” OR “chin osteotomy”) AND (“iliac crest” OR autogenous OR autograft* OR corticocancellous) WorldCat was used as a supplementary discovery source and therefore employed a shorter query based on the core concepts of genioplasty/chin advancement and autologous grafting. In the other databases, broader generic terms (including autogenous and autograft*) were combined with tissue-specific terms. Reference lists of eligible publications were also screened manually to identify studies involving graft types not explicitly named in every database query.

The reference lists of all publications considered eligible after full-text assessment were also examined manually to identify potentially relevant studies that had not been retrieved through the electronic searches. All records obtained from the databases and supplementary manual searching were exported to a single reference-management dataset. Duplicate records were removed before the screening process. To ensure that the search was sufficiently sensitive to capture the full range of autologous graft types, the graft block combined generic autograft terms (autogenous, autograft*, corticocancellous) with tissue-specific terms (iliac crest, adipose tissue, cartilage, dermal tissue, tooth). The generic terms were intended to retrieve records describing autologous graft types not named explicitly by a tissue-specific term, including coronoid process bone, external oblique line corticocancellous bone, mandibular bone harvested during orthognathic surgery, and osteocartilaginous composite grafts, all of which were represented among the included studies. Because a truncated term string was used in WorldCat and because no single query can name every possible donor site, the electronic search was supplemented by manual screening of the reference lists of all eligible full-text articles to identify any relevant study describing a graft type not explicitly captured by the database queries.

The remaining records were initially evaluated on the basis of their titles and abstracts. Full-text articles were retrieved when the available information indicated potential eligibility or when eligibility could not be determined conclusively from the title and abstract alone. Full texts were then assessed against the predefined inclusion and exclusion criteria. Only studies fulfilling all eligibility requirements and reporting original patient-level clinical outcomes were included in the qualitative synthesis.

### 2.5. Data Collection Process and Data Items

Study selection was undertaken by a six-member review team. Each title and abstract was independently assessed by two reviewers, with records allocated in pairs across the review team. Potentially eligible full texts were likewise independently assessed by two reviewers against the predefined eligibility criteria. Any disagreement between the two reviewers was resolved through discussion and consensus.

Data from the included studies were extracted using a standardized Microsoft Excel spreadsheet prepared before the data-collection process. The following information was recorded: first author, publication year, study design, clinical indication, sample size and patient characteristics, type and donor site of the autologous graft, surgical technique, comparator where applicable, follow-up duration, methods of outcome assessment, clinical and aesthetic outcomes, graft integration and stability, graft resorption, functional outcomes, complications, patient-reported outcomes, and the need for secondary procedures. The use of a predefined extraction form ensured that data were collected and organized consistently across all included studies.

### 2.6. Methodological Quality Assessment

The methodological quality and risk of bias of the included studies were independently evaluated by two reviewers, who were blinded to each other’s assessments. Because the review included different study designs, the appropriate design-specific Joanna Briggs Institute (JBI) critical appraisal checklist was applied to each publication. Accordingly, randomized clinical trials, quasi-experimental studies, observational studies, case series, and case reports were assessed using the corresponding JBI tools.

Depending on the study design, the appraisal covered the clarity of the relationship between the intervention and outcome, participant selection and comparability, the presence and appropriateness of a control group, consistency of care between groups, reliability and uniformity of outcome measurements, completeness of follow-up, identification of potential confounding factors, appropriateness of the statistical analysis, and completeness of clinical reporting. Each checklist item was classified as “yes,” “no,” “unclear,” or “not applicable,” in accordance with the JBI guidance. For descriptive reporting, the total number of items rated “yes” was presented together with the number of applicable checklist items. No thresholds for classifying studies as low, moderate, or high quality were prospectively defined in the protocol. Consequently, these raw totals were treated purely as a descriptive summary of how many appraisal criteria each study met; they were not converted into validated categories of methodological quality, were not used to derive a cut-off for inclusion or weighting, and should not be interpreted as an ordinal quality score, because the JBI checklists are not designed or validated to generate summary scores of this kind. Study-design labels were used to select the most appropriate design-specific JBI checklist and were not intended to imply methodological equivalence among heterogeneous clinical publications.

Disagreements between the two reviewers were resolved through discussion and consensus. Inter-rater agreement for the methodological assessment was determined using Cohen’s kappa statistic in MedCalc, version 23.1.7 (MedCalc Software Ltd., Brussels, Belgium). The resulting coefficient was 0.82 (*p* < 0.001), indicating near-perfect agreement between the reviewers.

## 3. Results

### 3.1. Study Selection

The database searches identified 795 records. After the removal of 337 duplicate records, 458 unique records remained and were screened on the basis of their titles and abstracts. Of these, 422 records were excluded because they did not meet the predefined eligibility criteria. The full texts of the remaining 36 publications were retrieved and assessed for eligibility. Nineteen full-text articles were subsequently excluded because they did not involve the use of an autologous tissue graft for chin augmentation, did not report relevant original patient-level outcomes, represented methodological or purely technical publications, or otherwise failed to satisfy the eligibility criteria. Ultimately, 17 unique clinical publications evaluating autologous tissue grafts for chin augmentation performed with or without genioplasty were included in the qualitative synthesis [1,3,5,6,11,12,15,38,57,58,59,60,61,62,63,64,65]. The complete selection process is presented in the PRISMA 2020 flow diagram in Figure 2.

### 3.2. General Characteristics of the Included Studies

The qualitative synthesis included 17 unique clinical publications evaluating autologous tissue grafts used for chin augmentation performed with or without genioplasty. The available evidence was methodologically heterogeneous and comprised one prospective randomized comparative trial, retrospective comparative and observational studies, clinical case series, case reports, and technique-oriented clinical reports presenting patient outcome data. The reported samples ranged from individual patients described in case reports to a large retrospective series of 567 patients who underwent 580 genioplasty procedures [3]. However, the approximately 200 procedures mentioned by Kerbrat et al. [57] represented the authors’ cumulative clinical experience rather than a formally defined study cohort.

The clinical indications varied considerably and included isolated chin deficiencies, such as insufficient chin projection, microgenia, and retrogenia; skeletal deficiencies requiring more extensive correction, including vertical chin deficiency and mandibular hypoplasia; and complex reconstructive conditions, including post-traumatic facial deformity, severe or syndromic mandibular retrognathia, deformities associated with temporomandibular joint ankylosis, and complex primary or secondary chin deficiencies [1,3,5,6,11,12,15,38,57,58,59,60,61,62,63,64,65]. Some interventions were performed as isolated chin augmentation or genioplasty, whereas others formed part of combined orthognathic, reconstructive, rhinoplasty, or temporomandibular joint surgery.

A broad spectrum of autologous materials was investigated. Soft-tissue augmentation involved autologous adipose tissue and dermal grafts [1,5,12,60]. Hard-tissue and structural augmentation included iliac crest bone [15,38,58,59], costal cartilage or costochondral rib grafts [3,6,59], coronoid process bone [61,62], external oblique line corticocancellous bone [65], mandibular corticocancellous bone harvested during intraoral vertical ramus osteotomy [63], and a tooth-derived graft prepared from an extracted third molar [57]. An osteocartilaginous graft obtained from the nasal hump during simultaneous rhinoplasty was also used [64]. In the series reported by Sabhlok et al. [61], only one of the 12 patients underwent additional chin augmentation; the remaining cases involved other maxillofacial reconstructive indications.

The surgical procedures and methods used to evaluate their outcomes varied considerably. Follow-up periods ranged from three months in an individual post-traumatic case [58] to a median of 58 months, with a range of 12–120 months, in the nasal hump graft series [64]. The main characteristics of the included publications are summarized in Table 1, while detailed information regarding the clinical indications, graft sources, surgical techniques, outcome assessments, stability, resorption, and complications is provided in Table 2.

### 3.3. Main Study Outcomes

Across the included studies, two conceptually distinct approaches were used: structural skeletal grafting (autologous bone and cartilage), intended to reposition or augment the bony chin and provide durable structural support, and soft-tissue/contour augmentation (autologous adipose and dermal grafts), intended to enhance chin contour and soft-tissue volume without altering the underlying skeletal base. Because these approaches differ in anatomical objective, biological mechanism, and the outcome measures used to evaluate them, findings for skeletal and soft-tissue grafts are presented separately below and should not be interpreted as directly comparable.

#### 3.3.1. Clinical and Aesthetic Outcomes

Clinical and aesthetic outcomes were evaluated using heterogeneous objective and subjective methods. Clinical examination and photographic documentation were the most frequently used assessment approaches [3,5,6,11,58,59,60,61,62,64,65]. Conventional radiographic evaluation, including lateral cephalometry and panoramic radiography, was used to assess skeletal position, bone healing, and hard- and soft-tissue changes [15,38,58,59,62]. Three-dimensional CT or CBCT imaging was applied for surgical planning and postoperative evaluation in selected studies [1,15,58,60,65]. Ultrasonography was used to measure changes in soft-tissue thickness and graft maintenance following adipose or dermal tissue augmentation [5,12]. Histological examination of transplanted adipose tissue was performed by Borisenko et al. [5], while Helmy et al. [1] used CBCT-based virtual planning and a three-dimensional injection guide to standardize treatment and evaluate postoperative soft-tissue changes.

Across the different clinical indications, most studies reported improvements in chin projection, facial profile, lower facial contour, symmetry, or overall facial proportions after treatment [1,3,5,6,11,12,15,38,57,58,59,60,62,63,64,65] irrespective of whether augmentation was achieved through structural skeletal grafting or soft-tissue/contour grafting; however, the specific parameters used to define ‘improvement’ differed between the two approaches, as detailed below. Quantitative outcome measures included soft-tissue thickness at the pogonion [5], vertical hard- and soft-tissue augmentation [38], dermal graft survival and resorption [12], three-dimensional skeletal and soft-tissue measurements [65], and vertical augmentation and bone resorption after grafting with mandibular corticocancellous bone [63].

Helmy et al. [1] reported significant improvements in soft-tissue pogonion position, mentolabial angle, facial convexity, and FACE-Q satisfaction scores after autologous fat augmentation, PEEK implantation, and sliding genioplasty. Although all three approaches improved chin appearance, greater soft-tissue relapse was observed in the autologous fat group at six months. This comparison illustrates the broader distinction between the two categories: because autologous fat grafting augments soft-tissue volume without providing rigid skeletal support, some degree of relapse relative to bone-based or alloplastic skeletal augmentation reflects an expected biological difference rather than inferior efficacy. Wang et al. [60] found that autologous fat grafting improved sagittal chin projection more effectively than vertical height, but did not provide quantitative measurements of graft retention or resorption.

Functional outcomes were reported mainly in patients treated for severe mandibular hypoplasia, post-traumatic deformity, syndromic retrognathia, or temporomandibular joint ankylosis. These outcomes included improvement or maintenance of occlusion, mandibular position, facial symmetry, mandibular function, and mouth opening [15,58,59,62]. Patient-reported outcomes were available in a limited number of studies and indicated improved satisfaction following autologous fat augmentation, dermal grafting, PEEK implantation, and sliding genioplasty [1,12,60].

#### 3.3.2. Graft Stability and Resorption

Clinical or radiographic stability was reported for autologous bone, cartilage, adipose tissue, dermal tissue, tooth-derived grafts, and osteocartilaginous grafts; however, ‘stability’ reflects fundamentally different biological processes depending on graft type. For structural skeletal grafts (bone, cartilage, tooth-derived, and osteocartilaginous grafts), stability refers to bone or cartilage union, incorporation, and maintenance of skeletal position. For soft-tissue/contour grafts (adipose and dermal tissue), stability instead refers to volumetric graft survival against expected resorption. The methods used to assess stability and the duration of follow-up also differed substantially between studies, further limiting direct comparison. Except where a standardized quantitative measurement is specified below, reports of maintained projection, integration, or absent resorption reflect qualitative clinical or radiographic observation and should not be read as quantitatively demonstrated dimensional stability.

Iliac crest bone grafts demonstrated satisfactory incorporation, bone union, and maintenance of the reconstructed chin or mandibular position [15,38,58,59]. Kim et al. [38] reported a mean vertical hard-tissue augmentation of 4.2 mm, accompanied by a 4.0 mm soft-tissue change, with no clinically evident vertical graft resorption reported. O’Connell et al. [15] observed uneventful graft incorporation and stable mandibular and chin positions after 34–36 months, with no clinical or radiographic evidence of resorption.

Coronoid process bone grafts were reported to maintain their position without clinically evident loss of projection; however, standardized quantitative resorption measurements were not reported [61,62]. Nevertheless, only one patient in the broader 12-patient series reported by Sabhlok et al. [61] underwent chin augmentation. External oblique line corticocancellous grafts showed satisfactory integration, stable skeletal and soft-tissue changes, and no clinically evident graft resorption during 10 months to 3 years of follow-up [65].

Kim et al. [63] reported mean vertical augmentation of 4.32 mm and mean vertical bone resorption of 0.39 mm, corresponding to approximately 9.02% of the initial augmentation at one year. The third-molar graft described by Kerbrat et al. [57] was reported to show graft integration and maintained augmentation during follow-up. However, the publication did not present a formally defined cohort or standardized quantitative measurements of graft resorption.

Autologous costal cartilage grafts maintained chin projection during follow-up, with no evidence of graft resorption in the series reported by Zhang et al. [6]. Stable clinical outcomes were also described after costochondral and iliac crest grafting by Fieldhouse et al. [59], although resorption was not measured quantitatively. Wolfe et al. [3] reported stable long-term correction following staged genioplasty and supplementary costal cartilage grafting but did not provide quantitative graft-volume measurements. Osteocartilaginous nasal hump grafts showed no reported displacement or resorption during a median follow-up of 58 months [64].

In contrast to the skeletal grafts described above, soft-tissue/contour grafts exhibited more variable volume maintenance, consistent with their different biological objective of augmenting contour rather than providing rigid structural support. Borisenko et al. [5] demonstrated persistent increases in soft-tissue thickness at the pogonion and histologically confirmed the presence of viable adipocytes with fibrous integration at 12 months. Soft-tissue thickness decreased after the initial postoperative increase, but the study did not report a formal percentage graft-survival or resorption rate. Helmy et al. [1] observed greater soft-tissue relapse at six months after autologous fat grafting than after PEEK implantation or sliding genioplasty. Wang et al. [60] reported satisfactory aesthetic improvement after fat grafting but did not quantify graft survival, volume retention, or resorption.

Kim et al. [12] provided a quantitative assessment of dermal graft maintenance. Mean graft resorption was 32.2% at six months and 34.9% at 12 months, corresponding to graft-survival rates of 67.8% and 65.1%, respectively. Most resorption occurred during the first six postoperative months, with relatively little additional volume loss thereafter.

#### 3.3.3. Safety and Complications

Major graft-related or procedure-related adverse events were not reported in several studies [1,6,12,15,57,59,61,64]. However, complication reporting was inconsistent, several publications involved only individual patients or small case series, and adverse events were not always collected systematically. Consequently, the incidence of serious complications cannot be estimated reliably from the available evidence. Denominators are reported below whenever they were available. The nature of reported complications also differed by graft category: neurological injury and donor-site morbidity were reported mainly in association with skeletal grafting and osteotomy-based procedures, whereas contour irregularities and the need for secondary lipofilling were reported specifically after soft-tissue/contour grafting, reflecting their distinct anatomical and biological mechanisms.

Neurological complications included five mental nerve injuries identified and repaired intraoperatively in the large retrospective series reported by Wolfe et al. [3]. Transient marginal mandibular nerve weakness occurred in the post-traumatic case described by Vajda et al. [58]. Temporary inferior alveolar nerve sensory deficits were reported in both patients treated by O’Connell et al. [15]. Transient mental nerve paraesthesia was observed after horizontal T-genioplasty [11]. Helmy et al. [1] reported mild, temporary chin or lower-lip paraesthesia in the PEEK and sliding genioplasty groups, with complete resolution.

Infectious complications included three late infections in early interpositional bone-graft cases reported by Wolfe et al. [3] and one postoperative infection requiring wire removal after horizontal T-genioplasty [11]. Cingi et al. [64] reported infection in 5 of 124 patients treated with osteocartilaginous nasal hump grafts; graft removal was required in two cases. Zhang et al. [6] reported persistent nasal-tip erythema in two patients following costal cartilage harvesting for combined rhinoplasty and chin augmentation. These events resolved following treatment, and no graft-related infections were reported.

Other recipient-site events included transient submental swelling or hematoma in three patients treated with iliac crest bone grafting, all of which resolved spontaneously [38]. Following subplatysmal fat grafting, contour irregularities developed in 14 of 77 patients (18%) and were corrected with secondary lipofilling [5]. Two patients who underwent dermal grafting received additional fat grafting to increase chin volume [12]. In the alloplastic implant group evaluated by Borisenko et al. [5], inflammatory complications, hematoma, and contour asymmetry were reported; these events should be distinguished from complications associated with autologous fat grafting.

Donor-site morbidity was infrequently reported. No donor-site infection or permanent gait disturbance occurred after iliac crest bone harvesting [38], and no clinically significant donor-site morbidity was reported after coronoid process grafting [62]. Borisenko et al. [5] identified scar retraction at the submental harvesting site as a potential concern, particularly in slender patients. No donor-site complications were reported after osteocartilaginous nasal hump grafting [64].

### 3.4. Quality Assessment

The included publications were appraised using the JBI checklists selected according to their reported study design: case report, case series, cohort study, or randomized controlled trial. The totals below represent only the number of checklist items rated “yes” out of those applicable, and are provided solely as a descriptive summary. Because no predefined thresholds were established, these counts should not be read as validated quality grades, as an ordinal ranking of the studies, or as directly comparable across the different design-specific checklists, which contain different numbers and types of items. Two publications were evaluated with the JBI Case Report Checklist. One met 7 of 8 criteria [58], while the other met 6 of 8 criteria [59].

Thirteen publications were evaluated with the JBI Case Series Checklist. The number of criteria rated “yes” was 10 for Cingi et al. [64]; 9 for references [15,65]; 8 for references [12,38,60,62,63]; 7 for Zhang et al. [6]; 6 for references [11,57]; and 5 for Wolfe et al. [3]. These totals are reported descriptively and do not constitute validated quality categories.

One cohort study met 7 of 11 applicable JBI criteria [5], and one randomized controlled trial met 7 of 13 criteria [1] (see Table 3).

## 4. Discussion

The present systematic review aimed to characterize the clinical applications and reported outcomes of autologous tissue grafts in patients undergoing chin augmentation with or without genioplasty. Comparative effectiveness was considered where applicable but was not required for inclusion [38,66,67,68,69]. The available evidence suggests that autologous grafts can produce clinically satisfactory outcomes when the graft type is selected according to the anatomical deficiency and the intended surgical correction. The available evidence suggests that autologous bone grafts obtained from the iliac crest, mandibular external oblique line, coronoid process, or bone removed during intraoral vertical ramus osteotomy may provide satisfactory integration and skeletal support, with limited clinically evident resorption reported [15,26,35,38,61,62,63,65,70,71,72,73,74,75,76]. Costal cartilage and costochondral grafts were also reported to maintain chin projection during follow-up [3,6,59], while a tooth-derived graft and an osteocartilaginous nasal hump graft were described as providing augmentation in selected clinical situations, although the available clinical evidence was limited [57,64]. In contrast, adipose and dermal grafts improved chin projection and patient satisfaction but showed less predictable volume maintenance, including soft-tissue relapse after fat grafting and approximately 35% dermal graft resorption after 12 months [1,5,12,60]. Although these findings support the clinical usefulness of autologous grafting, the limited number of comparative studies and the substantial methodological heterogeneity do not allow the superiority of autologous materials over sliding genioplasty or alloplastic implants to be established. This comparison should itself be interpreted cautiously: structural skeletal grafts (bone, cartilage) are the more direct biological counterpart to sliding genioplasty and rigid alloplastic implants, whereas soft-tissue/contour grafts (adipose, dermal tissue) address a different clinical goal and are not intended to substitute for skeletal repositioning.

The type and source of the autologous graft should be selected according to the character and extent of the deformity [35,60,77,78,79,80,81,82,83,84,85,86,87]. For clinical interpretation, these grafts can be broadly considered as soft-tissue volume grafts and structural skeletal grafts, reflecting their different biological functions and treatment goals. Among soft-tissue volume grafts, autologous adipose tissue may be suitable for mild or moderate sagittal deficiencies, soft-tissue contour irregularities, and age-related changes in the lower face because it can be delivered with relatively limited surgical invasiveness [1,5,60]. Its principal limitation is variable volume retention, which may lead to soft-tissue relapse, contour irregularities, or secondary lipofilling. Dermal grafts may provide an alternative for patients seeking moderate soft-tissue augmentation without osseous genioplasty or an alloplastic implant, although partial resorption should be anticipated [12]. By contrast, among structural skeletal grafts, autologous bone grafts may be considered for vertical augmentation, major skeletal advancement, large intersegmental defects, severe microgenia or retrognathia, and complex orthognathic reconstruction because they can provide structural support and facilitate osseous continuity [15,38,58,61,62,63,65]. This represents a clinically plausible pattern derived from limited, predominantly non-comparative evidence and should not be interpreted as a comparative evidence-based hierarchy of graft materials. Costal cartilage may be considered when substantial additional volume is required, particularly in complex, secondary, or combined rhinoplasty and chin augmentation procedures [3,6,59]. Donor-site morbidity is an important consideration in graft selection. Iliac crest harvesting can provide substantial graft volume but requires an additional surgical site, whereas mandibular, coronoid, external oblique line, tooth-derived, or nasal hump grafts may utilize tissue already exposed or removed during the primary procedure, potentially reducing additional donor-site morbidity. Tooth-derived and osteocartilaginous nasal hump grafts represent opportunistic donor materials, each supported by only a single included report [57,64]. They should therefore be regarded as selected, technique-specific options for carefully chosen patients in whom suitable tissue is already available, rather than as established alternatives of proven equivalence to bone or cartilage grafting.

The surgical technique may be as important as the biological properties of the selected graft. Successful outcomes depended on appropriate graft preparation, accurate adaptation to the recipient site, maintenance of vascularized soft-tissue attachments where applicable, and stable fixation [88,89,90,91,92,93,94,95,96,97,98]. Iliac crest bone grafts demonstrated satisfactory incorporation and maintained skeletal support when they were properly adapted and stabilized within genioplasty or mandibular osteotomy defects [15,38,59]. Similarly, external oblique line and coronoid process grafts showed stable integration following careful shaping and fixation [61,62,65]. Costal cartilage grafts were sculpted to reproduce the desired chin contour and fixed within a prepared recipient pocket, resulting in maintained projection without reported resorption [6]. In soft-tissue augmentation, Borisenko et al. [5] combined subplatysmal fat grafting with medial platysmaplasty and demonstrated persistent graft viability on histological examination. Helmy et al. [1] used virtual planning and a patient-specific three-dimensional guide to standardize fat placement. These findings suggest that outcome predictability cannot be attributed solely to graft composition and should also be considered in relation to graft processing, recipient-site preparation, fixation, surgical planning, and postoperative management.

Serious graft-related complications were not frequently reported, but their incidence could not be estimated reliably because adverse-event collection and reporting were inconsistent. The reported safety profile also differed according to the graft and surgical procedure. Temporary sensory disturbances were reported after osseous genioplasty and complex mandibular reconstruction, including mental, marginal mandibular, and inferior alveolar nerve dysfunction [1,3,11,15,58]. Most of these disturbances resolved during follow-up. Infectious complications were infrequently reported but occurred in early interpositional bone-graft cases, after horizontal T-genioplasty, and following osteocartilaginous nasal hump grafting [3,11,64]. In the latter series, infection occurred in 5 of 124 patients and required graft removal in two cases [64]. Autologous adipose tissue was not associated with infectious complications in the Borisenko study, although contour irregularities developed in 14 of 77 patients and required secondary lipofilling [5]. Two patients treated with dermal grafts also underwent additional fat augmentation to increase chin volume [12]. The comparative study by Helmy et al. [1] demonstrated improvement in patient satisfaction after fat grafting, PEEK implantation, and sliding genioplasty, but greater soft-tissue relapse occurred in the fat-grafting group. Consequently, autologous grafts should not be considered uniformly superior or complication-free; their clinical value depends on appropriate indication, careful technique, anticipated graft behavior, and a balanced assessment of recipient- and donor-site morbidity.

The findings of this review should be interpreted in light of several limitations. The included publications were highly heterogeneous with respect to study design, patient characteristics, clinical indications, graft materials, surgical techniques, outcome definitions, assessment methods, and follow-up duration. Much of the available evidence consisted of retrospective studies, small case series, case reports, or technique-oriented clinical descriptions, while controlled comparative evidence was limited. The inclusion of case reports and technique-oriented clinical publications increased the comprehensiveness of the review but limited the strength of the available evidence and warrants cautious interpretation of the findings. Case reports and very small case series were considered low-weight descriptive evidence because they provide limited evidence regarding effectiveness, safety, and long-term stability. Some publications included mixed maxillofacial reconstructive populations, with only a subset of patients undergoing chin augmentation, as in the series reported by Sabhlok et al. [61]. In addition, the approximately 200 procedures mentioned by Kerbrat et al. [57] did not constitute a formally defined study cohort. Graft stability and resorption were frequently described qualitatively, and standardized three-dimensional measurements and validated patient-reported outcome instruments were used only in selected studies. The limited and inconsistent use of three-dimensional assessment also restricts comparability between studies. Advances in CBCT-based virtual planning, surgical guides, and volumetric follow-up may improve surgical reproducibility and provide more standardized assessment of skeletal and soft-tissue outcomes in future studies. These differences precluded a meaningful quantitative meta-analysis and limited the strength of direct comparisons between materials. In addition, stability outcomes were assessed using fundamentally different methods across graft types. Cephalometric skeletal measurements cannot be directly compared with volumetric assessments of adipose or dermal grafts, further limiting comparability between studies. Consequently, findings for structural skeletal grafts and soft-tissue/contour grafts should be regarded as addressing different clinical questions rather than as directly comparable alternatives. Future research should include adequately powered prospective comparative studies with clearly defined indications, standardized grafting protocols, uniform follow-up intervals, and long-term three-dimensional assessment of graft volume, skeletal stability, and soft-tissue response. Studies should also incorporate validated patient-reported outcome measures, standardized reporting of recipient- and donor-site complications, and direct comparisons between autologous grafts, sliding genioplasty, and patient-specific alloplastic implants. Such evidence would facilitate the development of clinically useful recommendations for selecting the most appropriate graft and surgical technique for individual chin deformities. No formal framework for rating the certainty of the overall body of evidence, such as GRADE, was applied; therefore, the clinical implications should be regarded as descriptive and hypothesis-generating.

## 5. Conclusions

Autologous tissue grafts can provide clinically satisfactory aesthetic and functional outcomes in chin augmentation performed with or without genioplasty. The available evidence suggests that autologous bone grafts from the iliac crest and mandibular donor sites, as well as costal cartilage and costochondral grafts, may provide stable structural support, satisfactory integration, and limited clinically relevant resorption; however, these findings are based on limited and heterogeneous evidence. Tooth-derived and osteocartilaginous nasal hump grafts may represent selected, context-dependent options in carefully selected patients when suitable tissue is available during another planned procedure, although clinical evidence for these approaches remains limited. Autologous adipose and dermal grafts improved chin projection and contour with relatively limited invasiveness, although their volume maintenance was less predictable and secondary augmentation was occasionally required. Importantly, the available evidence does not establish the superiority of any single autologous graft material or technique over another; the reported differences reflect their distinct anatomical objectives and biological behavior rather than a demonstrated hierarchy of effectiveness.

Rather than being guided by any presumed superiority of one material, graft selection should be determined primarily by the anatomical requirements of the deformity (including the required augmentation volume and the need for skeletal versus soft-tissue augmentation), the availability of suitable donor tissue, the surgical context (including whether tissue is already exposed or removed during a concurrent procedure), and the anticipated recipient- and donor-site morbidity. The current evidence does not permit definitive conclusions regarding the superiority of autologous grafting over sliding genioplasty or alloplastic augmentation because most available publications were retrospective studies, small case series, case reports, or technique-oriented descriptions. Therefore, these findings should be interpreted cautiously until prospective comparative studies with standardized outcome assessment and long-term follow-up become available.

## Figures and Tables

**Table 1:** General characteristics of the included studies.

Author, Year	Aim of the Study	Materials and Methods	Results	Conclusion
Borisenko (2025) [5]	To compare chin augmentation using alloplastic implants with subplatysmal autologous fat grafting combined with medial platysmaplasty.	Retrospective comparative study of 170 patients: alloplastic implant group (n = 93) and subplatysmal fat autograft group (n = 77). Outcomes were evaluated clinically and photographically and, where applicable, using ultrasonography, CT, and histological examination during follow-up.	Both approaches improved chin projection. In the autograft group, ultrasonography showed increased soft-tissue thickness at the pogonion, and histology confirmed viable adipocytes with fibrous integration at 12 months. No infectious or inflammatory complications occurred in the autograft group; contour irregularities developed in 14 patients (18%) and were corrected with secondary lipofilling. The implant group was associated with inflammation, hematoma, and contour asymmetry.	Subplatysmal autologous fat grafting provided sustained aesthetic improvement with no infectious complications, although secondary contour correction was required in a minority of patients.
Vajda (2021) [58]	A case report of severe post-traumatic condylar resorption and facial skeletal deformity treated surgically without total joint replacement.	38-year-old female patient with right mandibular trauma (at age 2) presented with significant facial asymmetry, a 10 mm malocclusion, apertognathia, and an open bite on the left side. A CT scan confirmed hypoplasia of the right mandible and maxilla, as well as the absence of a condyle. Surgery: LeFort I osteotomy, left sagittal split osteotomy, right inverted-L ramus osteotomy, genioplasty, and anterior iliac crest bone grafting.	Facial symmetry and occlusion were substantially improved. Transient right marginal mandibular nerve weakness resolved by week 7. A 2 mm anterior open bite developed at 3 months, corrected with orthodontic intrusion. No infections or wound complications occurred.	Severe post-traumatic mandibular hypoplasia can be successfully corrected without condylar reconstruction when TMJ function is preserved. Inverted-L osteotomy combined with iliac bone grafting could be an alternative to total joint replacement.
Helmy (2024) [1]	This study aimed to assess changes in the soft-tissue profile following autologous fat augmentation or PEEK implantation compared with sliding genioplasty for correction of chin deficiency in patients with retruded chins.	33 patients with chin deficiency were enrolled and allocated into three groups: fat augmentation (intervention I), PEEK augmentation (intervention II), and osseous genioplasty as the control. All patients underwent preoperative and postoperative CBCT scans. Diagnostic workup, virtual planning, and evaluation were performed using MIMICS, 3-MATIC, and PROPLAN software. Patient satisfaction was assessed with the Face-Q questionnaire.	Fat augmentation showed significantly greater soft tissue relapse than control at 6 months (mean difference = 0.770), while PEEK did not differ from control (mean difference = −0.060). No group showed significant relapse changes across follow-up (fat *p* = 0.1389; PEEK *p* = 0.8739; control *p* = 0.8410). All groups showed significant improvement in chin satisfaction over time (fat *p* = 0.0165; PEEK *p* = 0.0150; control *p* = 0.0293).	Fat augmentation may represent an appropriate treatment option for patients with mild to moderate chin deficiency. PEEK PSI provides a stable surgical outcome.
Wolfe (2006) [3]	To review long-term experience with genioplasty and assess management of complex chin deficiencies requiring secondary autogenous augmentation.	Retrospective review of 580 genioplasties performed in 567 patients over 28 years, including cases managed with staged genioplasty and autogenous costal cartilage grafting.	Osseous genioplasty effectively corrected severe chin deformities and failed implant cases. Costal cartilage grafts provided additional augmentation when skeletal advancement alone was insufficient.	Autogenous costal cartilage grafting is a valuable adjunct after genioplasty in severe or multiply operated chin deficiencies, offering stable autologous augmentation.
Kim (2005) [38]	To evaluate long-term hard- and soft-tissue changes and graft stability following vertical height augmentation genioplasty with autogenous iliac bone graft.	Retrospective clinical and radiographic assessment of 23 patients treated with vertical augmentation genioplasty using interpositional autogenous iliac crest bone graft. Serial cephalometric analyses and follow-up examinations were performed up to ≥1 year postoperatively.	Mean vertical osseous augmentation was 4.2 mm and corresponding soft-tissue advancement was 4.0 mm, demonstrating high hard-to-soft tissue predictability. Grafted bone showed satisfactory union, maintained stability, and no significant resorption or major complications.	Vertical height augmentation genioplasty using autogenous iliac bone graft provides predictable soft-tissue response, stable bone integration, and reliable long-term chin augmentation.
Fieldhouse (1974) [59]	To report management of bilateral TMJ ankylosis associated with severe micrognathia and mandibular retrusion.	Case report of a 31-year-old female treated with bilateral resection of ankylotic masses, autogenous costochondral rib graft reconstruction, augmentation genioplasty using iliac bone graft, and subsequent chin onlay grafting.	Stable mandibular position and function were maintained during 2-year follow-up, with mouth opening of 3.5 cm and improved facial profile.	Combined autogenous costochondral grafting and augmentation genioplasty effectively restored mandibular function and corrected micrognathia in severe TMJ ankylosis.
Zhang (2019) [6]	The study evaluated clinical strategies for the use of autologous costal cartilage grafts in combined rhinoplasty and genioplasty procedures.	Autologous rib cartilage was harvested and shaped into a boat-shaped chin graft for genioplasty. The graft was inserted through an intraoral approach and stabilized with titanium or absorbable fixation nails.	The procedure resulted in satisfactory chin contour and facial profile improvement during 6–18 months of follow-up. No graft resorption was observed.	Autologous costal cartilage grafting appears to be an effective and stable option for chin augmentation with satisfactory aesthetic outcomes.
Kim (2016) [12]	To evaluate the clinical outcomes, graft survival, and resorption characteristics of augmentation genioplasty performed with a double-folded autologous dermal graft.	58 patients underwent augmentation genioplasty using a four-layered double-folded autologous dermal graft harvested from the gluteal region. Graft thickness and volume maintenance were evaluated by serial ultrasonographic examinations performed preoperatively and at 1, 6, and 12 months after.	The dermal graft demonstrated a survival rate of approximately 65% after 12 months, with an average resorption rate of 34.9%. Most patients (54/56 available for follow-up) were satisfied with the aesthetic outcome, and no major complications or graft-related adverse events were reported.	Double-folded autologous dermal grafting provided predictable and satisfactory outcomes for chin augmentation with a low complication rate.
Wang (2015) [60]	To evaluate the clinical outcomes of autologous fat grafting for chin augmentation and assess its effectiveness in improving chin projection.	Retrospective study of 105 patients with sagittal and/or vertical chin deficiencies treated with autologous fat grafting. Postoperative follow-up assessed aesthetic improvement, patient satisfaction, and the need for secondary procedures.	Most patients reported aesthetic improvement after fat grafting, with over half achieving marked improvement. Chin projection improved more effectively in the horizontal (sagittal) dimension than in vertical height. No major complications were observed.	Autologous fat grafting is a suitable option for selected patients seeking chin augmentation, mainly improving sagittal projection, while vertical lengthening remains limited.
Sabhlok (2014) [61]	This study aimed to assess the usefulness of autogenous coronoid process bone grafts in maxillofacial reconstructive surgery.	A 12-patient series (age 18–50, mean 34) used coronoid process grafts for maxillofacial reconstruction: 3 orbital defects, 1 blowout fracture, 1 anterior maxillary wall defect, 2 mandibular defects, 1 chin augmentation after horizontal flip genioplasty, 2 implant-site augmentations, and 2 TMJ ankylosis reconstructions. Technique: The coronoid is harvested intraorally via the external oblique ridge (ramus retractor, Kocher clamp, fissure bur, osteotome, diathermy release from temporalis) and closed with 4-0 Vicryl. Applications: orbital floor reconstruction (Weber-Ferguson approach, pedicled on temporalis, minimal resorption); blowout fracture repair as an iliac crest alternative; buccal plate recontouring (1.5 mm screws + graft material, screws removed at 6 months); small mandibular/zygomaticomaxillary defect bridging; and TMJ ankylosis (shaped to mimic a condyle, fixed with titanium screws), sometimes combined with chin augmentation after horizontal flip genioplasty.	All 12 reconstructions succeeded, with good graft take and no extrusion, infection, excessive resorption, or functional problems. Orbital cases showed no enophthalmos/diplopia; transient trismus occurred in 3/4 cases (resolved in 1–2 weeks). Implants were placed after 6 months (two-stage protocol) in the 2 mandibular augmentation cases; one required implant removal at 2 years due to local infection. Trauma-defect grafts restored good contour and function. TMJ ankylosis cases showed stable graft position on radiographs (no nonunion/reankylosis) with satisfactory mouth opening at 1 year (32 mm and 34 mm).	The mandibular coronoid process is a reliable autogenous donor site for moderate-sized maxillofacial defects and orbital floor repair. As an accessible intramembranous corticocancellous bone, it offers adequate volume, favorable morphology, and low resorption, with intraoral harvest avoiding visible scarring or major donor-site morbidity. Its properties suit orbital, condylar/ramus (TMJ ankylosis), and adjunctive chin reconstruction, making it a versatile option with predictable functional and aesthetic outcomes.
Jafarian (2014) [62]	The aim of this article was to introduce a new and versatile technique for chin augmentation that uses the resected hypertrophic coronoid process as an onlay bone graft in patients with TMJ ankylosis.	Two patients with bilateral TMJ ankylosis and microgenia underwent single-stage gap arthroplasty with interpositional temporalis fascia graft, bilateral coronoidectomy, and chin augmentation using the resected coronoid processes as autogenous grafts. TMJ access was via preauricular Al-Khayat-Bramley incision, with fascia interposed after resection of the ankylotic mass and coronoids. Chin augmentation used an intraoral genioplasty approach: the genial cortex was perforated (bur holes) and shaped coronoid grafts fixed to the mentum with titanium screws. Postoperative care included antibiotics, analgesia, early jaw physiotherapy, and annual clinical/radiologic follow-up.	Both patients tolerated the single-stage procedure well, with no major intraoperative or immediate postoperative complications. Satisfactory mouth opening was achieved after gap arthroplasty and maintained with physiotherapy throughout follow-up. Chin augmentation with autogenous coronoid grafts produced improved lower facial projection and correction of microgenia in both cases. Clinical and radiographic controls up to at least 6 months showed stable graft position, maintained joint function, and acceptable aesthetic outcomes.	Single-stage management of TMJ ankylosis with gap arthroplasty and interpositional temporalis fascia graft combined with chin augmentation using elongated coronoid process as an autogenous graft is a feasible and effective approach. In both cases, it simultaneously restored mandibular function and improved lower facial aesthetics without significant donor-site morbidity, suggesting that the coronoid process is a valuable and versatile graft source in this setting.
Zang (2024) [65]	To evaluate the effectiveness and aesthetic outcomes of genioplasty combined with autogenous external oblique line bone grafting for the correction of severe microgenia associated with a hamster-like facial contour.	Retrospective case series (n = 8) evaluating genioplasty with autogenous EOL grafting. Facial skeletal and soft-tissue changes were assessed by preoperative and long-term follow-up 3D-CT measurements and analyzed statistically using paired *t*-tests.	Eight female patients (mean age 24.8 years) were followed for 10 months to 3 years after surgery. Significant improvements were observed in mandibular width, lower facial proportions, chin projection, and lower lip position. No major complications, graft resorption, or impaired bone healing were reported during follow-up.	Genioplasty combined with autogenous external oblique line (EOL) bone grafting appears to be a safe and effective technique for correcting severe microgenia. The procedure enhances both frontal and lateral facial contours, improves chin projection and lower facial harmony, and provides a convenient intraoral donor site with low complication rates.
O’Connell (2018) [15]	Aim: To present and evaluate the orthognathic surgical management and outcomes in two sisters with Melnick-Needles syndrome undergoing bilateral mandibular ramus osteotomies combined with advancement genioplasty and iliac crest bone grafting to correct mandibular retrognathia.	Two adult sisters with Melnick-Needles syndrome presenting with mandibular hypoplasia and retrognathia underwent clinical and radiographic evaluation, including 3D CT to assess mandibular morphology and plan surgery. Both were treated with bilateral vertical mandibular ramus osteotomies via an extraoral submandibular approach, mandibular advancement into planned occlusion, and advancement genioplasty with autogenous iliac crest bone grafting to the ramus gaps and chin. Fixation was achieved with miniplates and monocortical screws. Standardized pre- and postoperative photographs and radiographs were obtained, with follow-up of 36 and 34 months, respectively.	Both sisters achieved improved mandibular projection, facial profile, and occlusion following bilateral ramus osteotomies with advancement genioplasty and iliac crest grafting. Radiographs confirmed stable mandibular advancement, graft incorporation, and maintained fixation at long-term follow-up (36 months in case 1, 34 months in case 2). The only postoperative complication in both cases was a temporary inferior alveolar nerve sensory deficit, which resolved over time. No relapse, infection, or hardware-related complications were observed.	Melnick-Needles syndrome is a rare skeletal dysplasia associated with severe mandibular hypoplasia and retrognathia, raising concerns about bone handling and healing. These two cases show that conventional orthognathic surgery using vertical mandibular ramus osteotomy and advancement genioplasty with monocortical fixation and iliac crest bone grafting can be performed safely, with satisfactory bone healing and stable functional and aesthetic outcomes, without the need for adjunctive BMP-2.
Grime (1990) [11]	To describe the horizontal-T genioplasty technique.	The technique was presented in 4 clinical cases. A horizontal bone segment is cut from the inferior border of the chin, preserving the lingual soft tissue attachment. The segment is then divided into three sagittal parts (not always equal) and repositioned in a horizontal “T” configuration by advancing the central and lateral fragments. Then the segments are fixed (any gaps can be filled with autogenous bone grafts or hydroxyapatite).	- effective augmentation - narrowing of the chin - Asymmetry correction - Good-long term stability - One infection reported	The technique is equal to conventional genioplasty.
Kerbrat (2021) [57]	To describe the surgical technique for augmentation (correcting vertical chin deficency) genioplasty using an extracted third molar as an autogenous graft.	After extraction of the third molar during orthognathic surgery, the crown was separated from the roots and the pulp was removed. Then the crown was shaped into a wedge, placed into the genioplasty osteotomy gap and stabilized with wires and/or screws. The augmentation height was determined on cephalometric analysis and clinical assessment.	- stable long-term outcomes with successful integration of the tooth graft - no increased risk of infection	The technique is a good alternative to iliac or calvaria bone graft. It provides stable results with low morbidity rate.
Kim (2020) [63]	To introduce VHAG technique (vertical height augmentation genioplasty) using autogenous bone achieved from IVRO (intraoral vertical ramus osteotomy).	The technique is presented on 12 patients. After conventional IVRO, the overhanging inferior portion of the proximal mandibular segment is resected, which allows the harvest of an autogenous corticocancellous bone block, that is then shaped and used during vertical height augmentation genioplasty (VHAG) to fill the gap between repositioned genial segments. The segments are stabilized with plates and can be supplemented with allogenic bone.	- mean augmentation 4.32 mm - mean resorption 0.39 mm (9.02%) - no major complications	- stable augmentation with low bone resorption - due to being useful for vertical microgenia with prognathism cases the technique requires more studies
Cingi (2010) [64]	To assess the usage of autogenous nasal hump transplantation in chin augmentation	The retrospective study included 124 patients (mean age 27 years) with prominent nasal humps and deficient chin projection. The patients underwent simultaneous rhinoplasty and mentoplasty, with a median follow-up of 58 months (range 12–120 months). The osteocartilaginous nasal hump was removed during rhinoplast, then reshaped into a chin graft and treated with gentamicin solution. Later the graft was implanted into a prepared mental pocket through either an intraoral or submental approach. The graft was stabilized within a pocket designed to provide support and minimize displacement. All procedures were performed under general anesthesia and by the same surgeon.	Satisfactory results in most patients. Five patients developed an infection (4%) patients, of whom two required graft removal (1.6%).	The technique may be a good and safe alternative for either patients with some form of microgenia or when the usage of alloplastic implants is not possible. The technique is relatively simple and reproducible by other surgeons.

**Table 2:** Detailed characteristics of the included studies.

Author, Year	Clinical Indication/Sample Size	Graft Type	Surgical Technique	Outcome/Follow-Up	Stability/Graft Resorption	Complications
Borisenko (2025) [5]	Insufficient chin projection with cervico-mental fat deposits and lower-face aging. Retrospective study, n = 170: implants group n = 93, subplatysmal fat autograft group n = 77.	Subplatysmal fat harvested intraoperatively from the cervico-mental (submental) compartment—no remote donor site.	Submental incision, medial platysmaplasty, intraoperative subplatysmal fat harvest, pocket creation in chin region, graft placement and fixation with absorbable sutures, layered closure.	Ultrasound (pogonion soft tissue thickness), CT (implant group), clinical photography, histology (punch biopsy, H&E, ×100) at 3, 6, 12 months. Main outcomes: chin projection gain (cm), soft tissue volume (mm), graft viability. Mean follow-up 18.3 months (range 2–43).	Ultrasound at pogonion: baseline 6.01 ± 1.8 mm → 14.33 ± 0.48 mm (3 mo) → 10.36 ± 0.5 mm (6 mo) → 8.9 ± 0.55 mm (12 mo). Chin projection gain: 0.8–1.2 cm (3 mo), 0.7–1.3 cm (6 mo), 0.6–1.9 cm (12 mo). Histology confirmed viable adipocytes with fibrous integration at 12 months.	No infectious or inflammatory complications. Contour irregularities in 14 patients (18%), corrected with lipofilling. Scar retraction at submental donor site noted as a risk in slender patients.
Vajda (2021) [58]	Post-traumatic condylar resorption with hemimandibular hypoplasia, severe facial asymmetry, and malocclusion (10 mm occlusal cant, left open bite, chin deviation). Case report, n = 1: 38-year-old female, childhood trauma to right mandible at age 2	Autogenous cortical block bone graft harvested from the right anterior iliac crest. Used to reconstruct the right mandibular ramus and right zygomaticomaxillary buttress.	LeFort I osteotomy + right inverted-L ramus osteotomy (transcervical) + left BSSO (transoral) + genioplasty, with iliac crest onlay/block graft (reconstruction plate, bicortical screws) augmenting the deficient right ramus. Planned via Virtual Surgical Planning; no TMJ reconstruction performed.	Clinical examination, cephalometry (A-P and lateral), panoramic radiographs, CT, and photographic documentation. Main outcomes: facial symmetry, occlusion, nerve function. Follow-up: 3 months.	Not reported. At 3 months, occlusion was intact with symmetric bilateral molar contacts. A 2 mm anterior open bite developed postoperatively, corrected by orthodontic posterior intrusion. No graft resorption data provided.	Transient right marginal mandibular nerve weakness, resolved by week 7. Postoperative 2 mm anterior open bite, corrected orthodontically. No infection, wound breakdown, or graft failure reported.
Helmy (2024) [1]	Prospective randomized controlled trial including 33 adult patients (mean age 25.5 ± 3.5 years; 10% male, 90% female) with mild sagittal chin deficiency (grade 1 retrogenia ≤10 mm, isolated under-projected bony chin without major maxillofacial deformity), allocated to three groups: autogenous fat augmentation (n = 11), onlay PEEK patient-specific implant (n = 11), and sliding osseous genioplasty (control, n = 11).	In this study, chin augmentation was performed using three different approaches: autogenous fat grafting with processed abdominal lipoaspirate injected in a supraperiosteal plane, an alloplastic onlay patient-specific PEEK implant fixed to the mandibular symphysis, and sliding osseous genioplasty in which the mandibular symphysis segment was advanced and fixed without the use of additional graft material.	All patients had CBCT-based virtual planning with 3D guides/implants. Fat: Abdominal liposuction (2 mm cannula, 50-cc syringe), filtered/emulsified through a 2 mm filter, injected supraperiosteally via 2 ports using a 3D-printed volume guide indexed to the anterior teeth. PEEK: Patient-specific implant designed from CBCT data (virtual genioplasty, fixation-hole planning), milled from PEEK, steam-sterilized; placed via transoral vestibular incision, fixed with 2.0 mm titanium screws, closed in two layers. Control: Sliding osseous genioplasty using 3D-printed cutting/positioning guides; horizontal osteotomy with microsaw, chin segment advanced per template and fixed with titanium microscrews, closed in layers.	All 33 patients completed 6-month follow-up with uneventful healing and no major complications. All three techniques significantly improved soft-tissue pogonion projection, mentolabial angle, and facial convexity. FACE-Q satisfaction improved significantly in all groups, with no differences between techniques. Soft-tissue relapse at 6 months was greatest in the fat group (partial resorption), while PEEK and sliding genioplasty remained stable.	At 6-month follow-up, the autogenous fat group showed significant soft-tissue relapse consistent with partial fat resorption, whereas the PEEK implant and sliding osseous genioplasty groups demonstrated stable soft-tissue pogonion, mentolabial angle, and facial convexity, indicating minimal or no clinically relevant resorption or positional change in the underlying bone/implant.	No significant intraoperative or immediate postoperative complications occurred in any group. In the PEEK and sliding genioplasty groups, patients experienced postoperative edema lasting 2–3 weeks and mild transient paresthesia of the chin and lower lip, which resolved completely by the end of the second week; no infections or implant/genioplasty failures were reported. In the fat group, edema was minimal, with no ecchymosis and no neurosensory disturbances, and all cases healed uneventfully.
Wolfe (2006) [3]	Severe developmental or syndromic retrogenia, failed alloplastic chin implants, multiply operated chin; retrospective review; 567 patients, 580 genioplasties. Subgroup analysis of staged genioplasty and salvage procedures.	Costal cartilage graft (rib); additionally cranial bone grafts and vascularized iliac osteocutaneous free flap in selected cases.	Osseous genioplasty alone or staged genioplasty; costal cartilage onlay graft fixed to symphysis through submental approach when further augmentation was required.	Clinical assessment, chart review, operative records and photographic evaluation. Improvement of chin projection and correction of severe chin deficiency reported. Follow-up varied; illustrative cases up to 8 years.	Stable chin correction reported after staged genioplasty; costal cartilage graft described as resistant to infection, displacement and bony erosion. No quantitative resorption data reported.	Five mental nerve injuries repaired intraoperatively; three late infections in early interpositional bone-graft cases; hardware removal in 14 additional patients
Kim (2005) [38]	Vertical chin deficiency; retrospective clinical and radiographic study; n = 23. Four cases included simultaneous advancement genioplasty; 14 underwent two-jaw surgery	Autogenous iliac crest corticocancellous bone graft (iliac bone graft, IBG).	Horizontal symphyseal osteotomy with interpositional iliac bone graft for vertical height augmentation genioplasty (VHA	Lateral cephalometry, panoramic radiographs and clinical follow-up. Hard- and soft-tissue changes assessed preoperatively and up to ≥1 year postoperatively. Mean vertical augmentation: 4.2 mm (hard tissue) and 4.0 mm (soft tissue); hard-to-soft tissue ratio 1:0.96.	Stable genial segment position; normal bony union and graft integration. No significant vertical graft resorption detected up to 1 year after hardware removal.	No infection or wound dehiscence. Three cases of transient submental swelling/hematoma resolved spontaneously. No donor-site infection or permanent gait disturbance.
Fieldhouse (1974) [59]	Bilateral TMJ ankylosis with severe micrognathia and mandibular retrusion; case report	Costochondral rib grafts (6th and 7th ribs), iliac crest bone grafts.	Bilateral ankylosis release with costochondral graft reconstruction; augmentation genioplasty using interpositional iliac bone graft; secondary iliac crest onlay graft for additional chin projection.	Clinical and radiographic assessment over 2-year follow-up evaluated mandibular position, chin projection, and mouth opening. Incisal opening improved to 3.5 cm, with maintained mandibular function and profile improvement.	Position and function of the mandible remained stable during 2-year follow-up. No quantitative graft resorption data reported.	No major postoperative complications reported; postoperative course described as satisfactory.
Zhang (2019) [6]	Clinical case series including 28 female patients (mean age 24.5 years) presenting with varying degrees of nasal and chin underdevelopment or deformity.	Autologous costal cartilage graft harvested through an inframammary incision.	Costal cartilage was sculpted into a boat-shaped graft for chin augmentation. Through an intraoral approach, the periosteum was elevated to expose the mentum, and the graft was placed in the prepared chin space and secured with titanium or absorbable nails.	Outcomes were assessed by clinical exam and aesthetic evaluation of facial contour and chin appearance, with 21 patients completing 6–18 months of follow-up.	Stable augmentation was maintained throughout the 6–18 months follow-up, with no evidence of cartilage resorption.	Two patients developed persistent nasal tip skin redness, which resolved after treatment. No other complications were reported.
Kim (2016) [12]	Microgenia and deficient chin projection in patients unwilling to undergo osseous genioplasty or alloplastic implantation. Case series including 58 patients (15 men, 43 women; mean age 29.4 years).	Autologous dermal graft (double-folded, four-layered dermis) harvested from the gluteal region adjacent to the intergluteal crease.	Intraoral augmentation genioplasty using a supraperiosteal pocket. A double-folded dermal onlay graft was placed over the chin and secured to the periosteum to increase chin projection.	Graft thickness and volume were assessed by serial ultrasonography at 1, 6, and 12 months, evaluating graft survival, resorption, chin projection maintenance, and patient satisfaction (54/56 satisfied). Follow-up ranged from 12 months to 3 years.	Mean graft resorption was 32.2% at 6 months and 34.9% at 12 months (survival rates 67.8% and 65.1%, respectively), with resorption peaking in the first 6 months and remaining stable thereafter.	No major complications were reported. Two patients underwent secondary fat grafting to increase chin volume.
Wang (2015) [60]	Retrospective study of 105 patients with mild sagittal/vertical chin deficiencies: microgenia (n = 64), unsatisfactory outcome after mandibular advancement for retrognathia (n = 11), secondary augmentation after alloplastic implant removal (n = 16), and chin augmentation for facial balance after rhinoplasty (n = 14).	Autologous fat graft harvested from the lower abdomen, flank, lateral thigh or inner thigh.	Percutaneous multilayer fat grafting with placement of processed autologous fat into the subcutaneous and submuscular chin compartments to enhance sagittal projection and, in selected patients, augment vertical chin height.	Outcomes were assessed by objective evaluation and patient self-assessment. Main outcomes included improvement in chin projection and patient—reported improvement. Mean follow-up was 21 months (range: 7–37 months).	Not reported. No quantitative assessment of graft survival, volume retention, or graft resorption was provided; however, sagittal augmentation appeared more effective than vertical augmentation.	No major complications reported.
Sabhlok (2014) [61]	12-patient series (age 18–50, mean 34) using autogenous coronoid process grafts for maxillofacial reconstruction. Indications: 3 orbital defects (extended maxillectomy), 1 blowout fracture, 2 TMJ ankylosis reconstructions, 1 chin augmentation after horizontal flip genioplasty, 1 anterior maxillary wall trauma defect, 2 mandibular defects, 2 implant-site bone augmentations.	Autogenous intramembranous cortico-cancellous bone graft harvested from the mandibular coronoid process	The coronoid process is exposed via an intraoral incision along the external oblique ridge, engaged with a fork ramus retractor and Kocher clamp, sectioned at its base with a fissure bur, and separated with an osteotome. Temporalis tendon attachments are released with diathermy while twisting the coronoid free with the clamp; muscle attachments are cleared before transfer to the recipient site. Closure is with 4-0 Vicryl.	All 12 cases succeeded: good graft uptake, no extrusion/infection/excessive resorption. Orbital: no enophthalmos/diplopia, transient trismus in 3/4 (1–2 wks). Implants (n = 2): integrated by 6 months; 1 later removed at 2 years (infection). TMJ: stable grafts, 32/34 mm mouth opening at 1 year.	Coronoid grafts showed good stability with minimal resorption; no excessive resorption, extrusion, or contour loss occurred, and pedicled applications (e.g., orbital floor reconstruction) maintained position over follow-up.	No major graft-related complications occurred (no extrusion, infection, nonunion, reankylosis, or excessive resorption). Transient trismus occurred in 3/4 orbital cases (resolved in 1–2 weeks). One trauma patient required implant removal at 2 years due to localized infection, not attributed to graft failure.
Jafarian (2014) [62]	The report describes 2 patients with long-standing bilateral temporomandibular joint ankylosis associated with retrognathia, microgenia, and lower facial deficiency, treated with single-stage gap arthroplasty, bilateral coronoidectomy, and simultaneous chin onlay augmentation using the elongated coronoid processes.	Autogenous onlay cortical bone graft using resected elongated mandibular coronoid processes harvested during bilateral coronoidectomy.	Single-stage, general anesthesia: bilateral TMJ gap arthroplasty (Al Khayat-Bramley incisions, ankylotic mass resected, temporalis fascia interposed), then bilateral coronoidectomy via the same access. Chin augmentation: intraoral incision (mental nerves protected), symphyseal cortex perforated, coronoids shaped/wired as onlay graft, fixed with titanium screws. Postop: antibiotics/analgesia, physiotherapy from day 5–7.	Both patients showed improved function and aesthetics, with maintained mouth opening and corrected microgenia/facial deficiency. Follow-up (3, 6 months, annually) showed stable chin projection with no reankylosis, resorption, or donor-site morbidity.	Coronoid onlay grafts showed stable chin augmentation with no significant loss of projection or contour; given their membranous, cortical nature, resorption was expected to be limited, and no clinically relevant graft failure occurred.	No major complications related to the coronoid onlay grafts or donor sites were reported. There was no mention of infection, graft exposure, fracture, or failure, nor of facial nerve injury or significant sensory disturbance. No reankylosis of the TMJ was observed during follow-up.
Zang (2024) [65]	G-EOL genioplasty corrects microgenia/retrogenia and “hamster-like” facial contour, improving lateral width and chin projection together. Candidates prefer autologous bone, have adequate EOL volume/quality on 3D-CT, and lack surgical contraindications. Retrospective series: 8 females (age 21–32, mean 24.8), G-EOL May 2019–Jul 2021, follow-up 10 months–3 years. 3D-CT bony data for all 8; soft-tissue CT for 6 (2 excluded, no BMI change). No short- or long-term complications observed.	Autogenous cortico-cancellous bone graft harvested from the mandibular external oblique line (EOL).	Intraoral “V” vestibular incision exposes the mandibular body under general anesthesia. 3D-CT-guided EOL osteotomy (ultrasonic osteotome, first molar ±5 mm anterior, antegonial notch posterior, ~5 mm below alveolar ridge, <3.2 mm lingual depth); bone trimmed into blocks. Horizontal genioplasty osteotomy mobilizes the chin (pedicle/nerve protected), advanced/rotated and fixed with titanium plates/screws. EOL blocks placed around osteotomy gaps and sutured; closure with drain, hardware removable after ≥12 months.	Follow-up: 10 months–3 years (3D-CT all 8; soft-tissue CT 6). No complications. Significant gains: bony width (B-MBP 77.85 → 71.99 mm, *p* < 0.001), chin projection (Pog-SPr 16.25 → 10.47 mm, *p* < 0.001), soft-tissue ratio (SMBP/MF 0.54 → 0.52, *p* = 0.042), lip protrusion (R-LL 7.18 → 2.16 mm, *p* = 0.001). Bone healing/graft stability satisfactory; hardware removed ≥12 months.	Follow-up (10 months–3 years) showed satisfactory bone healing and stability, with no significant graft resorption. Autogenous EOL corticocancellous grafts (membranous, high BMD) showed stable integration with maintained bony width/chin projection gains on 3D-CT; no implant loss or clinically relevant volume loss occurred.	G-EOL genioplasty is an effective, reliable technique for correcting microgenia and hamster-like lower-face contours, providing simultaneous gains in anteroposterior chin projection and transverse width, with good bone healing, long-term stability, minimal resorption, and low complications in this series.
O’Connell (2018) [15]	Severe mandibular hypoplasia/retrognathia with Melnick-Needles syndrome (OSA, tracheostomy-dependent in one case) treated with orthognathic advancement + iliac crest grafting over distraction osteogenesis, given abnormal bone architecture, increased collagen, hypodontia, and limited access. Sample: 2 female siblings (27, 21), bilateral ramus osteotomies + advancement genioplasty + iliac crest grafting; follow-up 34–36 months.	Autogenous iliac crest cortico-cancellous bone graft used to bridge ramus osteotomy gaps and augment the advancement genioplasty.	Bilateral extraoral submandibular incisions exposed the mandibular ramus. Vertical ramus osteotomy (sigmoid notch to inferior border, posterior to antilingula) advanced the mandible to planned occlusion, with iliac crest grafts filling the gaps and fixed via 2.0 mm miniplates/screws (drains placed). Transoral genioplasty then advanced the chin ~10 mm, secured with a 1.7 mm plate and augmented with iliac crest bone. Closure in layers with standard postop airway management.	Both sisters showed uneventful healing with stable mandibular advancement/chin position at 34–36 months. No major complications occurred beyond temporary inferior alveolar nerve sensory deficit. Occlusion, facial profile, and mandibular projection improved and were maintained long-term.	Over 34–36 months, iliac crest grafts bridging the ramus osteotomy gaps and genioplasty showed uneventful incorporation and stable mandibular/chin position, with no significant resorption or impaired healing despite Melnick-Needles-associated abnormal bone architecture.	Both patients had only a temporary inferior alveolar nerve sensory deficit, which resolved; no infection, non-union, fracture, or graft failure occurred. The elder sister’s permanent tracheostomy was maintained for psychological reasons, not as a surgical complication.
Grime (1990) [11]	Broad, flat, retruded or asymmetrical chin; severe microgenia; mandibular asymmetry. Case study. n = 4	Autologous bone graft from the inferior border of the chin.	Modified horizontal-T genioplasty: chin osteotomy divided into three segments; central segment and lateral limbs advanced and repositioned to achieve chin advancement, narrowing, and to correct asymmetry	Mainly clinical evaluation and photographic assessment. Outcomes: chin advancement, contour improvement, correction of asymmetry, more esthetic facial appearance. Longest reported follow-up: 4 years.	Stable results reported clinically.	One postoperative infection requiring wire removal. Transient mental nerve paraesthesia (recovery within 12 weeks). No wound dehiscence. No major complications
Kerbrat (2021) [57]	Vertical chin deficiency. Technique-oriented clinical report based on approximately 200 procedures performed over 20 years; no formally defined study cohort was presented.	Autologous third molar tooth graft(harvested during orthognathic surgery).	Augmentation genioplasty with horizontal symphyseal osteotomy. Third molar crown shaped into a wedge and placed as an interpositional graft within the osteotomy gap, fixed with wires and/or screws.	-	Stable long-term results and complete integration of the tooth graft into the bone.	No notable complications reported. Low morbidity and no infection reported.
Kim (2020) [63]	Microgenia accompanied by mandibular prognathism. Case study. n = 12	Autogenous corticocancellous bone graft harvested from vertically overhanging proximal mandibular segments during intraoral vertical ramus osteotomy (IVRO).	After conventional IVRO, the proximal segments are repositioned laterally and excess bone extending below the mandibular border is removed to prevent relapse and postoperative open bite. The resected bone can be harvested as an autogenous corticocancellous graft. The harvested bone is trimmed according to the surgical plan, placed between the bone segments to increase chin height, and stabilized with fixation plates.	CBCT with 3D reconstruction performed preoperatively and postoperatively at 1 month and at 1 year. The models were assessed by the vertical changes of the chin with reference to a horizontal plane.	The mean (SD) amount of height Augmentation: 4.32 (1.40) mm The mean amount of vertical bone resorption: 0.39 (0.30) mm (9.02%)	No specific postoperative complication.
Cingi (2010) [64]	Microgenia with poor chin projection and large nasal hump undergoing simultaneous rhinoplasty. Retrospective case series. n = 124	Autogenous osteocartilaginous nasal hump graft.	Nasal hump removed during rhinoplasty, shaped into a chin graft, inserted through intraoral or submental approach into a prepared mental pocket and stabilized by periosteal support.	Clinical and photographic assessment of aesthetic outcome, infection, displacement, and complications. Median follow-up 58 months (range 12–120 months).	No graft displacement or resorption reported.	Infection: 5/124 with the need for 2 graft removals No graft necrosis/migration, bone erosion or donor-site complications reported.

**Table 3:** Quality Assessment of Included Studies.

Case Reports													
Author	Were the patient’s demographic characteristics clearly described?		Was the patient’s history clearly described and presented as a timeline?		Was the current clinical condition of the patient on presentation clearly described?		Were diagnostic tests or assessment methods and the results clearly described?		Was the intervention(s) or treatment procedure(s) clearly described?	Was the post-intervention clinical condition clearly described?	Were adverse events (harms) or unanticipated events identified and described?		Does the case report provide takeaway lessons?
Vajda [58]	Yes		No		Yes		Yes		Yes	Yes	Yes		Yes
Fieldhouse [59]	Yes		Yes		No		Yes		Yes	No	Yes		Yes
Case Series													
Author	Were there clear criteria for inclusion in the case series?	Was the condition measured in a standard, reliable way for all participants included in the case series?	Were valid methods used for the identification of the condition for all participants included in the case series?		Did the case series have consecutive inclusion of participants?	Did the case series have a complete inclusion of participants?	Was there clear reporting of the demographics of the participants in the study?		Was there clear reporting of the clinical information of the participants?	Were the outcomes or follow-up results of cases clearly reported?	Was there clear reporting of the presenting site(s)/clinic(s) demographic information?		Was statistical analysis appropriate?
Wolfe [3]	No	Yes	Yes		Yes	No	No		Yes	Yes	No		No
Kim [38]	Yes	Yes	Yes		Yes	Yes	No		Yes	Yes	No		Yes
Zhang [6]	Yes	Yes	Yes		Yes	No	Yes		Yes	Yes	No		No
Kim [12]	Yes	Yes	Yes		Yes	Yes	Yes		Yes	Yes	No		Yes
Wang [60]	Yes	Yes	Yes		Yes	Yes	No		Yes	Yes	No		Yes
Sabhlok [61]	Yes	No	Yes		Yes	Yes	Yes		Yes	Yes	No		No
Jafarian [62]	Yes	Yes	Yes		Yes	Yes	No		Yes	Yes	No		Yes
Zang [65]	Yes	Yes	Yes		Yes	Yes	Yes		Yes	Yes	No		Yes
O’Connell [15]	Yes	Yes	Yes		Yes	Yes	Yes		Yes	Yes	No		Yes
Grime [11]	Yes	No	Yes		Yes	Yes	No		Yes	Yes	No		No
Kerbrat [57]	No	No	Yes		Yes	Yes	Yes		Yes	Yes	No		No
Kim [63]	Yes	Yes	Yes		Yes	Yes	No		Yes	Yes	No		Yes
Cingi [64]	Yes	Yes	Yes		Yes	Yes	Yes		Yes	Yes	Yes		Yes
Cohort Studies													
Author	Were the two groups similar and recruited from the same population?	Were the exposures measured similarly to assign people to both exposed and unexposed groups?	Was the exposure measured in a valid and reliable way?		Were confounding factors identified? Were strategies to deal with confounding factors stated?	Were strategies to deal with confounding factors stated?	Were the groups/participants free of the outcome at the start of the study (or at the moment of exposure)?		Were the outcomes measured in a valid and reliable way?	Was the follow-up time reported and sufficient to be long enough for outcomes to occur?	Was the follow up complete, and if not, were the reasons for the loss of follow-up described and explored?	Were strategies to address incomplete follow-up utilized?	Was appropriate statistical analysis used?
Borisenko [5]	Yes	No	Yes		No	No	Yes		Yes	Yes	Yes	No	Yes
Randomized Controlled Trials													
Author	Was true randomization used for the assignment of participants to treatment groups?	Was allocation to treatment groups concealed?	Were treatment groups similar at the baseline?	Were participants blind to treatment assignment?	Were those delivering the treatment blind to treatment assignment?	Were treatment groups treated identically other than the intervention of interest?	Were outcome assessors blind to treatment assignment?	Were outcomes measured in the same way for treatment groups?	Were outcomes measured in a reliable way?	Was follow-up complete, and if not, were differences between groups in terms of their follow-up adequately described and analyzed?	Were participants analyzed in the groups to which they were randomized?	Was appropriate statistical analysis used?	Was the trial design appropriate and were any deviations from the standard RCT design accounted for in the conduct and analysis of the trial?
Helmy [1]	Yes	No	Yes	Yes	No	Yes	No	Yes	Yes	Yes	Yes	Yes	Yes

## Data Availability

No new data were created or analyzed in this study. Data sharing is not applicable to this article.

## Supplementary Materials

The following supporting information can be downloaded at: https://www.mdpi.com/article/10.3390/jcm15187025/s1, Supplementary Table S1. PRISMA 2020 Main Checklist; Supplementary Table S2. Full-text studies excluded after eligibility assessment and reasons for exclusion.
