# Supplementary material for: Autologous Tissue Grafts for Chin Augmentation With or Without Genioplasty: A Systematic Review
Source: J Clin Med. 2026 Sep 10;15(18):7025. doi: 10.3390/jcm15187025 (PMC13607103; doi:10.3390/jcm15187025)
Supplement: Supplementary file 1 [file jcm-15-07025-s001.zip › jcm-4503304-supplementary.pdf]

Supplementary Table S1. PRISMA 2020 Main Checklist.

Topic	No.	Item	Location where item is reported
**TITLE**			
**Title**	1	Identify the report as a systematic review.	1
**ABSTRACT**			
**Abstract**	2	See the PRISMA 2020 for Abstracts checklist	1
**INTRODUCTION**			
**Rationale**	3	Describe the rationale for the review in the context of existing knowledge.	2-4
**Objectives**	4	Provide an explicit statement of the objective(s) or question(s) the review addresses.	4-5
**METHODS**			
**Eligibility criteria**	5	Specify the inclusion and exclusion criteria for the review and how studies were grouped for the syntheses.	6-7
**Information sources**	6	Specify all databases, registers, websites, organisations, reference lists and other sources searched or consulted to identify studies. Specify the date when each source was last searched or consulted.	7-8
**Search strategy**	7	Present the full search strategies for all databases, registers and websites, including any filters and limits used.	7-8
**Selection process**	8	Specify the methods used to decide whether a study met the inclusion criteria of the review, including how many reviewers screened each record and each report retrieved, whether they worked independently, and if applicable, details of automation tools used in the process.	8
**Data collection process**	9	Specify the methods used to collect data from reports, including how many reviewers collected data from each report, whether they worked independently, any processes for obtaining or confirming data from study investigators, and if applicable, details of automation tools used in the process.	8
**Data items**	10a	List and define all outcomes for which data were sought. Specify whether all results that were compatible with each outcome domain in each study were sought (e.g. for all measures, time points, analyses), and if not, the methods used to decide which results to collect.	8
	10b	List and define all other variables for which data were sought (e.g. participant and intervention characteristics, funding sources). Describe any assumptions made about any missing or unclear information.	n/a
**Study risk of bias assessment**	11	Specify the methods used to assess risk of bias in the included studies, including details of the tool(s) used, how many reviewers assessed each study and whether they worked independently, and if applicable, details of automation tools used in the process.	8

Topic	No.	Item	Location where item is reported
**Effect measures**	12	Specify for each outcome the effect measure(s) (e.g. risk ratio, mean difference) used in the synthesis or presentation of results.	n/a
**Synthesis methods**	13a	Describe the processes used to decide which studies were eligible for each synthesis (e.g. tabulating the study intervention characteristics and comparing against the planned groups for each synthesis (item 5)).	6-7
	13b	Describe any methods required to prepare the data for presentation or synthesis, such as handling of missing summary statistics, or data conversions.	7
	13c	Describe any methods used to tabulate or visually display results of individual studies and syntheses.	7
	13d	Describe any methods used to synthesize results and provide a rationale for the choice(s). If meta-analysis was performed, describe the model(s), method(s) to identify the presence and extent of statistical heterogeneity, and software package(s) used.	n/a
	13e	Describe any methods used to explore possible causes of heterogeneity among study results (e.g. subgroup analysis, meta-regression).	n/a
	13f	Describe any sensitivity analyses conducted to assess robustness of the synthesized results.	n/a
**Reporting bias assessment**	14	Describe any methods used to assess risk of bias due to missing results in a synthesis (arising from reporting biases).	7
**Certainty assessment**	15	Describe any methods used to assess certainty (or confidence) in the body of evidence for an outcome.	n/a
**RESULTS**			
**Study selection**	16a	Describe the results of the search and selection process, from the number of records identified in the search to the number of studies included in the review, ideally using a flow diagram.	9
	16b	Cite studies that might appear to meet the inclusion criteria, but which were excluded, and explain why they were excluded.	n/a
**Study characteristics**	17	Cite each included study and present its characteristics.	23-25
**Risk of bias in studies**	18	Present assessments of risk of bias for each included study.	16-22
**Results of individual studies**	19	For all outcomes, present, for each study: (a) summary statistics for each group (where appropriate) and (b) an effect estimate and its precision (e.g. confidence/credible interval), ideally using structured tables or plots.	16-22
**Results of syntheses**	20a	For each synthesis, briefly summarise the characteristics and risk of bias among contributing studies.	n/a
	20b	Present results of all statistical syntheses conducted. If meta-analysis was done, present for each the summary estimate and its precision (e.g. confidence/credible interval) and measures of statistical heterogeneity. If comparing groups, describe the direction of the effect.	n/a

Topic	No.	Item	Location where item is reported
	20c	Present results of all investigations of possible causes of heterogeneity among study results.	n/a
	20d	Present results of all sensitivity analyses conducted to assess the robustness of the synthesized results.	n/a
**Reporting biases**	21	Present assessments of risk of bias due to missing results (arising from reporting biases) for each synthesis assessed.	23
**Certainty of evidence**	22	Present assessments of certainty (or confidence) in the body of evidence for each outcome assessed.	n/a
**DISCUSSION**			
**Discussion**	23a	Provide a general interpretation of the results in the context of other evidence.	25-26
	23b	Discuss any limitations of the evidence included in the review.	26-27
	23c	Discuss any limitations of the review processes used.	26-27
	23d	Discuss implications of the results for practice, policy, and future research.	26-27
**OTHER INFORMATION**			
**Registration and protocol**	24a	Provide registration information for the review, including register name and registration number, or state that the review was not registered.	5
	24b	Indicate where the review protocol can be accessed, or state that a protocol was not prepared.	n/a
	24c	Describe and explain any amendments to information provided at registration or in the protocol.	n/a
**Support**	25	Describe sources of financial or non-financial support for the review, and the role of the funders or sponsors in the review.	28
**Competing interests**	26	Declare any competing interests of review authors.	28
**Availability of data, code and other materials**	27	Report which of the following are publicly available and where they can be found: template data collection forms; data extracted from included studies; data used for all analyses; analytic code; any other materials used in the review.	n/a

PRIMSA Abstract Checklist

Topic	No.	Item	Reported?
**TITLE**			
**Title**	1	Identify the report as a systematic review.	Yes
**BACKGROUND**			
**Objectives**	2	Provide an explicit statement of the main objective(s) or question(s) the review addresses.	Yes
**METHODS**			
**Eligibility criteria**	3	Specify the inclusion and exclusion criteria for the review.	No
**Information sources**	4	Specify the information sources (e.g. databases, registers) used to identify studies and the date when each was last searched.	Yes
**Risk of bias**	5	Specify the methods used to assess risk of bias in the included studies.	No
**Synthesis of results**	6	Specify the methods used to present and synthesize results.	No
**RESULTS**			
**Included studies**	7	Give the total number of included studies and participants and summarise relevant characteristics of studies.	Yes
**Synthesis of results**	8	Present results for main outcomes, preferably indicating the number of included studies and participants for each. If meta-analysis was done, report the summary estimate and confidence/credible interval. If comparing groups, indicate the direction of the effect (i.e. which group is favoured).	Yes
**DISCUSSION**			
**Limitations of evidence**	9	Provide a brief summary of the limitations of the evidence included in the review (e.g. study risk of bias, inconsistency and imprecision).	No
**Interpretation**	10	Provide a general interpretation of the results and important implications.	Yes
**OTHER**			
**Funding**	11	Specify the primary source of funding for the review.	No
**Registration**	12	Provide the register name and registration number.	No

From: Page MJ, McKenzie JE, Bossuyt PM, Boutron I, Hoffmann TC, Mulrow CD, et al. The PRISMA 2020 statement: an updated guideline for reporting systematic reviews. MetaArXiv. 2020, September 14. DOI: 10.31222/osf.io/v7gm2. For more information, visit: www.prisma-statement.org [44].

Supplementary Table S2. Full-text studies excluded after eligibility assessment and reasons for exclusion.

Author, year	Reason for exclusion
Chou & Wicaksono, 2025 [S1]	Conference abstract/poster only
Barreto et al., 2025 [S2]	Conference abstract/poster only
Al-Nakishbandi, 2025 [S3]	Conference abstract/poster only
Carlino, 2024 [S4]	Conference abstract/poster only
Bhatt et al., 2023 [S5]	Conference abstract/poster only
Lobato et al., 2023 [S6]	Methodological comparison of outcome-assessment methods
Carlino, 2020 [S7]	Conference abstract/poster only
Helmy et al., 2024 [S8]	Duplicate record of the included Helmy et al. (2024) study
García-Jiménez et al., 2013 [S9]	Conference abstract/poster only
Santanche & Bonarrigo, 1995 [S10]	Review article; no original clinical outcome data
Newton, 1973 [S11]	Review/technical overview; no original clinical outcome data
Borisenko et al., 2025 [S12]	Duplicate/overlapping study population with the included Borisenko et al. (2025) study
Rohrich et al., 2018 [S13]	Technique-focused article without extractable original clinical outcome data
Badr et al., 2016 [S14]	Literature review with an accompanying diagnostic case report; no eligible treatment-outcome data
Guyuron & Kadi, 1997 [S15]	Review/technical article; no eligible original clinical outcome data
Shi et al., 2020 [S16]	Animal study
Li et al., 2007 [S17]	Chinese-language publication
Luo et al., 2004 [S18]	Chinese-language publication
Zhang et al., 2002 [S19]	Chinese-language publication

References to studies excluded after full-text assessment

S1. Chou, P.; Wicaksono, D. Bone Grafting in Orthognathic Surgery: My Personal Experiences and Literature Reviews. Int. J. Oral Maxillofac. Surg. 2025, 54 (Suppl. 1), 247–248. https://doi.org/10.1016/j.ijom.2025.04.671.

S2. Barreto, V.; González-Jiménez, A.; Vieira Sebe, N.; Guerrero-Martín, M.; Mosteiro, C.; Roig, M.; Ranz-Colio, Á.; Baranda-Manterola, E.; Haddad-Riesgo, A.; Lago-Beack, B.; Pallarés, K.S.; Sánchez-Jáuregui Alpañés, E.; Eslava, J.M.; Sanz, J.A. Our Experience over the Past 5 Years with Patient Specific Implants in Maxillofacial Surgery. Int. J. Oral Maxillofac. Surg. 2025, 54 (Suppl. 1), 368. https://doi.org/10.1016/j.ijom.2025.04.981.

S3. Al-Nakishbandi, H. Orthognathic Surgery in Craniofacial Patients at a Tertiary Maxillofacial Centre: A 15-Year Retrospective Review of Surgical Interventions and Trends. Int. J. Oral Maxillofac. Surg. 2025, 54 (Suppl. 1), 13. https://doi.org/10.1016/j.ijom.2025.04.045.

S4. Carlino, F. Labiomental Graft to Flatten a Too Deep Fold after Advancement Genioplasty: Revision of the Technique after 5 Years of Clinical Outcomes. Int. J. Oral Maxillofac. Surg. 2024, 52 (Suppl. 2), 130.

S5. Bhatt, P.; Brader, T.; Lowe, M.; Turvey, T.A. Pediatrics, Craniofacial and Orthognathic Surgery Track: Stock vs. Custom Hardware for Virtually Planned Orthognathic Surgery: Analysis Involving Cost and Duration of Surgery. J. Oral Maxillofac. Surg. 2023, 81, e8.

S6. Lobato, R.C.; Camargo, C.P.; Buelvas Bustillo, A.M.; Ishida, L.C.; Gemperli, R. Volumetric Comparison between CT Scans and Smartphone-Based Photogrammetry in Patients Undergoing Chin Augmentation with Autologous Fat Graft. Aesthet. Surg. J. 2023, 43, NP310–NP321. https://doi.org/10.1093/asj/sjac335.

S7. Carlino, F. Too Deep Labio-Mental Fold after Large Advancement Genioplasty: A Proposal for Its Effective Correction. J. Oral Maxillofac. Surg. 2020, 78, e81.

S8. Helmy, A.; Shaker, M.A.; Fetouh, A.H.A.; Kadry, W.; Sadek, K. Evaluation of Soft Tissue Profile Changes Following Autogenous Fat or Onlay PEEK Augmentation versus Sliding Genioplasty for Correction of Deficient Chin: Randomized Controlled Clinical Trial. J. Stomatol. Oral Maxillofac. Surg. 2024, 125, 101939. https://doi.org/10.1016/j.jormas.2024.101939.

S9. García-Jiménez; Colmenero-Ruíz, C.; Rosón-Gómez, S.; Encinas-Bascones, A. Inverted L Osteotomy: Indications and Techniques. Int. J. Oral Maxillofac. Surg. 2013, 42, 1327. https://doi.org/10.1016/j.ijom.2013.07.541.

S10. Santanchè, P.; Bonarrigo, C. Mentoplastica additiva [Augmentation mentoplasty]. Plastica 1995, 27, 167–179.

S11. Newton, R.A. Mentoplasty. Can. J. Otolaryngol. 1973, 2, 235–237.

S12. Borisenko, A.S.; Sharobaro, V.I.; Burkhonova, N.S.; Avdeev, A.E.; Anurov, M.V.; Fisun, A.O.; Lysenko, N.A. Minimally Invasive Method for Harmonizing Insufficient Chin Projection Using Subplatysmal Fat Autografts. Plast. Surg. Aesthet. Med. 2025, 3, 12–21.

S13. Rohrich, R.J.; Sanniec, K.; Afrooz, P.N. Autologous Fat Grafting to the Chin: A Useful Adjunct in Complete Aesthetic Facial Rejuvenation. Plast. Reconstr. Surg. 2018, 142, 921–925. https://doi.org/10.1097/PRS.0000000000004817.

S14. Badr, F.F.; Mintline, M.; Ruprecht, A.; Cohen, D.; Blumberg, B.R.; Nair, M.K. Long-Standing Chin-Augmenting Costochondral Graft Creating a Diagnostic Challenge: A Case Report and Literature Review. Imaging Sci. Dent. 2016, 46, 279–284. https://doi.org/10.5624/isd.2016.46.4.279.

S15. Guyuron, B.; Kadi, J.S. Problems Following Genioplasty: Diagnosis and Treatment. Clin. Plast. Surg. 1997, 24, 507–514. https://doi.org/10.1016/S0094-1298(20)31042-7.

S16. Shi, L.; Tee, B.C.; Cotter, L.; Sun, Z. Enhance Mandibular Symphyseal Surface Bone Growth with Autologous Mesenchymal Stem Cell Sheets: An Animal Study. Aesthetic Plast. Surg. 2020, 44, 191–200. https://doi.org/10.1007/s00266-019-01494-3.

S17. Li, H.C.; Gui, L.; Lv, C.S.; Zhang, Z.Y.; Liu, Y.F.; Zhang, J. Comparison Study of Three Methods for the Treatment of Microgenia. Zhonghua Zheng Xing Wai Ke Za Zhi 2007, 23, 402–404. PMID: 18161355. [Article in Chinese].

S18. Luo, J.C.; Gui, L.; Zhang, Z.Y.; Teng, L.; Niu, F.; Jin, G.; Xia, D.L. Chin Augmentation with Bone Transplantation from the Mandible. Zhonghua Zheng Xing Wai Ke Za Zhi 2004, 20, 104–105. PMID: 15334929. [Article in Chinese].

S19. Zhang, Z.; Gui, L.; Teng, L.; Hou, Q. Surgical Management of Bilateral Mandibular Angle Prominence Associated with Microgenia. Zhonghua Zheng Xing Wai Ke Za Zhi 2002, 18, 214–216. PMID: 12382571. [Article in Chinese].
